# ReSkmer: modeling repeats allows *k*-mer-based alignment-free methods to calculate population genomic distances

**DOI:** 10.1186/s13059-026-04108-9

**Published:** 2026-05-23

**Authors:** Eduardo Charvel, Isaac Thomas, Homère J. Alves Monteiro, Shahab Sarmashghi, Glenn Dunshea, Vineet Bafna, Siavash Mirarab

**Affiliations:** 1 Department of Electrical & Computer Engineering, University of California, San Diego, La Jolla, USA; 2 Bioinformatics & Systems Biology Graduate Program, University of California, San Diego, La Jolla, USA; 3 Department of Computer Science & Engineering, University of California, San Diego, La Jolla, USA; 4 Center for Evolutionary Hologenomics, GLOBE Institute, University of Copenhagen, Copenhagen, Denmark; 5 Broad Institute of MIT and Harvard, Boston, Massachusetts USA; 6 Department of Natural History, NTNU University Museum, Norwegian University of Science and Technology, Trondheim, 7491 Norway

## Abstract

Understanding ecological change requires robust, inexpensive methods for monitoring populations. One approach is using low-coverage genome sequencing (genome skimming) to compute genetic diversity via assembly-free, alignment-free, *k*-mer-based methods to compute genomic distances from shotgun sequences using the intersection of *k*-mer sets. Skmer extended this approach to genome skims by modeling coverage and error. However, these methods ignore genome repetitiveness, hampering population genetic distance calculation. Here, we mathematically derive the expected intersection size between *k*-mers sampled from two repetitive genomes, accounting for repeats, coverage, and errors, leading to the method ReSkmer. Our experiments show highly accurate distances despite sampling highly repetitive genomes.

**Supplementary Information:**

The online version contains supplementary material available at 10.1186/s13059-026-04108-9.

## Introduction

As anthropogenic pressure continues to severely disrupt global ecosystems in recent years, biodiversity faces unprecedented levels of change and loss [1–5]. An important feature of loss of biodiversity and extinction is the loss of genetic diversity [6], which sustains the persistence of natural populations [7]. Genome-wide genetic diversity is an important gauge for assessing population genetic health [8], making it the basis for one of six broad categories of “essential biodiversity variables” used for detecting biodiversity change [9]. Moreover, falling costs have made it possible to shotgun sequence a reference sample for at most $10 per Gbp [10].

Methods like ANGSD [11] have been developed to provide measures of genetic diversity despite low coverage [12], given that a genome is available as the reference for mapping reads. However, assembling and finishing a reference genome remains expensive ($$$10^4$$–$$10^5$$ per genome). At the current pace, it may take decades before a comprehensive set of reference genomes exists to support ongoing, global monitoring of genetic diversity. Moreover, sequencing tends to be biased [13, 14], leaving many habitats underfunded and underrepresented in databases [15]. Thus, methods that enable the rapid and inexpensive assessment of population genetic metrics without reliance on reference genomes are increasingly valuable for biodiversity monitoring. One particularly promising avenue is obtaining low-coverage “genome skims” and using results to compare samples. These genome skims, which are far less expensive than high-coverage data, also hold promise for studying biodiversity at a reduced cost despite our inability to assemble full nuclear genomes from them [16].

The genomic distance between two genomes is a direct measure of genetic diversity ($\theta$), and previous work has considered if reference-free, alignment-free methods can estimate distance using only *k*-mers. Alignment-free sequence comparison methods have a long history, including methods that use spaced words, feature frequency profiles, length distribution [17–21], and *k*-mers [22–25]. The most widely used of these methods is perhaps Mash [26]. It generates an unbiased min-hash [27] sketch of an input genome skim, which allows it to compute the Jaccard index (*J*) between *k*-mer sets efficiently. It then estimates distance using [23] $d=1-\left( {}^{2J\!}/_{\!J+1}\right) ^{1/k}$ and also removes *k*-mers that appear once to alleviate the impact of sequencing errors. However, Mash is not designed for low-coverage data where many *k*-mers from a genome will not be sampled, resulting in distance overestimation [28].

The problem of distance calculation from low-coverage skims has been addressed by Skmer [28]. For each skim $\mathcal {S}^{(j)}$, Skmer uses its *k*-mer frequency spectrum to estimate three parameters – $\lambda ^{(j)}$ (*k*-mer coverage), $\epsilon ^{(j)}$ (sequencing error rate), and $L^{(j)}$ (genome length); based on these, it computes the probability that each genomic *k*-mer is sampled without error ($\eta ^{(j)}$), and the total number of *k*-mers generated from a position ($\zeta ^{(j)}$), as detailed in Table 2 (Methods section). Let the intersection and union of *k*-mers from genome skims $\mathcal {S}^{(1)}$ and $\mathcal {S}^{(2)}$ be *I* and *U*. For $L=L^{(1)}=L^{(2)}$, under certain assumptions (e.g., absence of repetitive *k*-mers), we can derive:

1 $$\begin{aligned} \mathbb {E}[|I|] \approx L \eta ^{(1)} \eta ^{(2)} (1 - d) ^ k \end{aligned}$$

and $\mathbb {E}[|U|]\approx L\zeta =L(\eta + \lambda (1 - (1 - \epsilon ) ^ k))$. Skmer uses Mash to compute the Jaccard index $J=\frac{|I|}{|U|}$ between $\mathcal {S}^{(1)}$ and $\mathcal {S}^{(2)}$ and estimates the distance *d* using:

2 $$\begin{aligned} 1-\left( \frac{2 (\zeta ^{(1)} L^{(1)} + \zeta ^{(2)} L^{(2)}) J}{\eta ^{(1)}\eta ^{(2)} (L^{(1)} + L^{(2)}) (1 + J)}\right) ^{1/k} \end{aligned}$$

which also corrects for different genome sizes. Note that this equation matches the original Mash distance if we set $L^{(1)}=L^{(2)}$ and $\eta ^{(j)}=\zeta ^{(j)}=1$, which would be the case if $\epsilon ^{(j)}=0$ and $\lambda ^{(j)}\rightarrow \infty$.

Skmer has proven accurate in benchmarking [30] and has been used extensively on real data [31–38] to increase the resolution of species identification [39–41]. However, its use has primarily been limited to interspecific phylogenetic applications rather than for intraspecific population genetics analyses, even though both uses are theoretically feasible. A key limitation of Skmer, like all *k*-mer-based distance calculation methods, is that it operates under the simplifying assumption that the underlying genomes are not repetitive. Let $r_i$ be the number of *k*-mers that appear *i* times in a genome (i.e., its repeat spectrum). The uniqueness ratio – the ratio of the number of unique *k*-mers to the length of the genome ($\textit{UR}=r_1/L$) – indicates what proportion of genomes made of *k*-mers that appear once. The UR is highly variable across genomes, ranging from 1.0 to as low as 0.28 [29]. When genomes are highly repetitive, Skmer’s methods for calculating sequencing parameters and translating *k*-mer similarity to distance both become inaccurate. We addressed both aspects to design ReSkmer.

In this paper, we introduce and evaluate a new method called ReSkmer. ReSkmer explicitly accounts for the impact of repetitive sequences in computing the distance between two species. We tested the accuracy of ReSkmer computations on extensive simulations as well as genome skims obtained from multiple species. Our results suggest that ReSkmer greatly improves the resolution of distances, allowing us to estimate population-level distances, making the method suitable for less studied species without the need for genome assembly and mapping.

## Results

### ReSkmer: repeats change the interpretation of Jaccard index

**Fig. 1:**
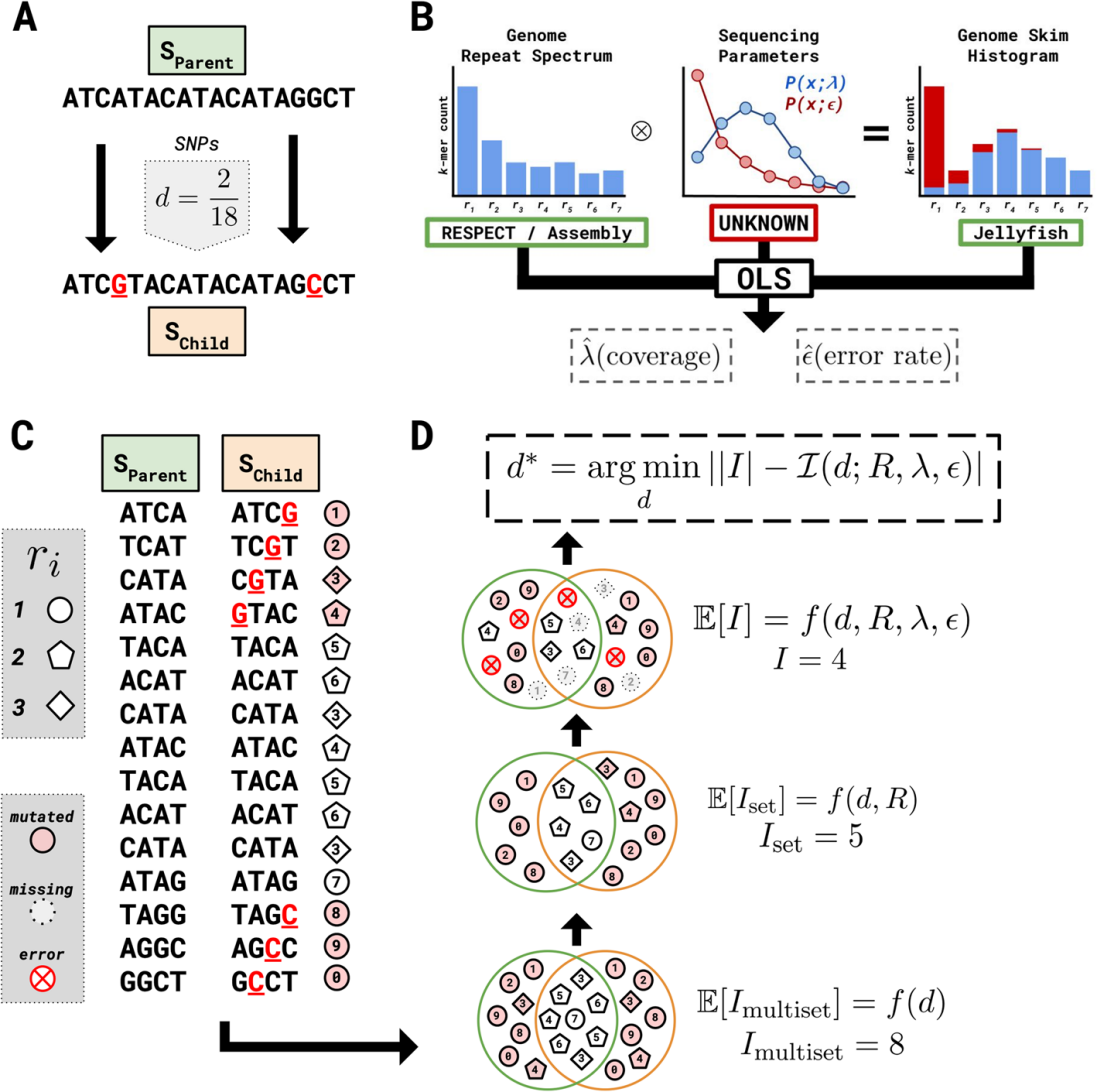
Effect of repeats and skimming on Mash Intersection. **A** SNPs are introduced to genomic sequences with repeats at a rate $\theta$. **B** The observed *k-*mer histogram is a function of a DNA sample's true repeat spectrum (*R*) and the sequencing process. Although the sequencing parameters are unknown, we can infer them using ordinary least squares when *R* is known. **C** Genomic sequences can be decomposed into *k*-mers that occur at different multiplicities (shown with different shapes). **D** If each sequence is expressed as multisets of *k*-mers, then the intersection of these multisets will only be a function of the genomic distance. However, if expressed as a set (e.g. Mash), then *k*-mers that appear multiple times in the sequences are collapsed into a single representative, meaning that for sequences with repeated *k-*mers, $I_{\text {set}} \ne I_{\text {multiset}}$. The intersection of two genome skims is further changed by coverage ($\lambda$) and sequencing error ($\epsilon$). Examples include the removal of *k*-mers #4 and #7 from the intersection because they were not covered without error. Meanwhile, at a rate related to repetitiveness, closely related *k*-mers may become the same due to error and increase the intersection. In order to get an accurate estimate of $\theta$, we must approximate the observed intersection using independent estimates of $\lambda$, $\epsilon$, and *R*

Modeling the evolution of repeats is challenging as repeats can arise from various biological processes, including transposons, duplication events, and variable tandem repeats. Currently, we lack reliable statistical models of change in repeat spectra across deep evolutionary time. However, for shallower time scales such as the evolution of populations or closely related species, we can work with a ‘parent child’ model (Fig. 1A). In this model, we consider a parent genome that accumulates mutations with some small probability *d* to generate a ‘child’ genome. New repeats do not arise; SNPs tend to break apart existing repeats (Additional file 1: Fig. S1).

ReSkmer takes as input two genome skims (collections of *k*-mers sampled from the genomes) and outputs pairwise estimates of genomic distance *d*. At population genetic scales, this parameter *d* is an estimate of the classic diversity parameter, $\theta$. Thus, ReSkmer executes three steps: 1) compute sequencing parameters for each skim, 2) compute the intersection between *k-*mer sets, 3) estimate the distance from the intersection and parameters (Fig. 1B-D).

#### Parameter estimation.

A listing of all parameters is provided in Table 2. A key parameter is a representative genome repeat spectrum ($R=r_1, \ldots r_m$) for the two individuals, where $r_i$ denotes the number of *k*-mers that occur exactly *i*-times in the genome. Related to *R* is the *observed* *k*-mer frequency histogram $O=[o_1,o_2,\ldots ]$ (Fig. 1B), where $o_i$ denotes the number of distinct *k*-mers observed exactly *i* times in the genome skim. *O* is a function of the repeat spectrum (*R*) of the underlying genome plus the stochastic sequencing process, encompassing both the random number of times a *k*-mer is covered – modeled as a Poisson distribution with parameter $\lambda$ – and whether a sequenced *k*-mer is erroneous – modeled as a Binomial with parameters $k,\epsilon$. Jointly estimating *R*, $\lambda$, and $\epsilon$ from the observed spectrum is challenging (an ill-conditioned problem [29]) without external information; however, a method called RESPECT is able to estimate *R* with reasonable accuracy using biologically realistic constraints and empirical data [29]. Alternatively, when a reference genome is available, its repeat spectrum *R* can be calculated directly (e.g., using Jellyfish [42]). Given *R*, we can compute $\lambda$ (Methods: Estimating genomic parameters section). Finally, using *R* and $\lambda$, we model $o_i$’s as a function of $\epsilon$ and use ordinary least squares (OLS) to infer a point estimate of $\epsilon$.

#### Estimating distance

With highly repetitive genomes, the Skmer equation, Eq. (2), no longer accurately estimates distance. In repetitive genomes, *k-*mers will occur at different multiplicities, and because set intersection ignores those multiplicities, Eq. (1) fails to hold (see Fig. 1C, D). Our main theoretical contribution is careful modeling of how repetitive *k*-mers change the intersection of *k*-mer sets obtained from error-prone samples of genomes (see Modeling the number of shared *k*-mers section). We modeled two events ignored by Skmer: 1) Erroneous *k-*mers can appear in the intersection not due to homology but sequencing error, thereby artificially inflating the intersection. 2) Repetitive *k*-mers may mutate in some copies and not in others. Thus, more repetitive *k-*mers are more likely to be in the intersection (Fig. 1D). We have derived the expectation of intersection size as a function of distance *d* for given parameters $R, \lambda , \epsilon$, culminating in a complex equation:

3 $$\begin{aligned} \mathbb {E}&[|I|] = \mathcal {I} (d;{R}^{(1)},\lambda ^{(1)},\epsilon ^{(1)},\eta ^{(1)},\lambda ^{(2)},\epsilon ^{(2)},\eta ^{(2)}) \approx \nonumber \\&\sum \limits _{i=1}^{m} r_i^{(1)}\left( 1-\left( 1-\eta {^{(1)}}\right) ^i \right) \left( 1-\left( 1-\eta ^{(2)}(1-d)^k\right) ^i\right) + 3k r_i^{(1)} \left( 1- e^{- i b \lambda ^{(1)} \epsilon ^{(1)} (1-\epsilon ^{(1)})^{k-1} } \right) \nonumber \\&\quad \left( 1-\left( (1-d)^k e^{ - b \lambda ^{(2)} \epsilon ^{(2)} (1-\epsilon ^{(2)})^{k-1}} - bd(1-d)^{k-1} \eta ^{(2)}+ \left( 1- (1-d)^k \right) \right) ^i\right) \end{aligned}$$

where $b= k (e^{-{}^{1\!}/_{\!3k}}-1)\approx {}^{-1\!}/_{\!3}$ is a constant.

Having this function $\mathcal {I}(d)$, we estimate *d* by finding:

4 $$\begin{aligned} d^* = \arg \underset{d}{\min }\ \left\lvert |I|- \mathcal {I}(d;{R}^{(1)},\lambda ^{(1)},\epsilon ^{(1)},\eta ^{(1)},\lambda ^{(2)},\epsilon ^{(2)},\eta ^{(2)}) \right\rvert \;. \end{aligned}$$

We approximate |*I*| using MinHash as implemented in Mash [26] to efficiently obtain a Jaccard index (*J*) for each pair of samples, which can then be used to estimate $|I| = {}^{J\!}/_{\!(1 + J)}(|\mathcal {S}^{(1)}| + |\mathcal {S}^{(2)}|)$. We solve the optimization problem using the hyperbolic Brent method [43]. Note that Brent requires that the difference between |*I*| and $\mathcal {I}(d)$ changes sign between the minimum and maximum *d* allowed (0 and 1). Additional file 1: Figure S2 shows that $\mathcal {I}(d)$ is monotonic for reasonable parameters (though this is not guaranteed). As long as |*I*| is inside the range determined by the parameters, the Brent method can find the root. If $|I|>\mathcal {I}(0)$, it cannot do so, and we set the distance to 0.

Plotting Eq. (1) versus Eq. (3) for various parameters (Additional file 1: Fig. S3), we see that the two equations can diverge substantially under some conditions. The divergences are small for low coverage and low repeat content, but increase if genomes become more repetitive or the coverage becomes high (e.g., $>10\times$). Skmer addressed the issue with high coverage for non-repetitive genomes by removing singleton *k*-mers when coverage is above 5$\times$, a trick that ReSkmer does not need to employ.

### Simulation results

We compare accuracy on skims simulated from six assemblies (Additional file 1: Table S1) chosen to represent a variety of repetitiveness as measured by their uniqueness ratios (UR $= r_1/L$). For each genome, we use the procedures explained in Simulation procedures section to simulate a variety of distances (0.1% to 20%), and explore the effects that different combinations of parameters ($\lambda$, *R*, and $\epsilon$) have on each of the methods. To focus on the resolution of small distances, we evaluate the error using the log error $\log _2(\hat{d}/d^*)$ where $\hat{d}$ and $d^*$ are estimated and true distances, respectively, replacing $-\infty$ with $-4$. We compare Mash and Skmer to ReSkmer with spectra obtained from a reference assembly (ReSkmer-ref) or with reference-free estimate from skims using RESPECT (ReSkmer-noref).

#### ReSkmer improves distances for high repeat inputs

**Fig. 2:**
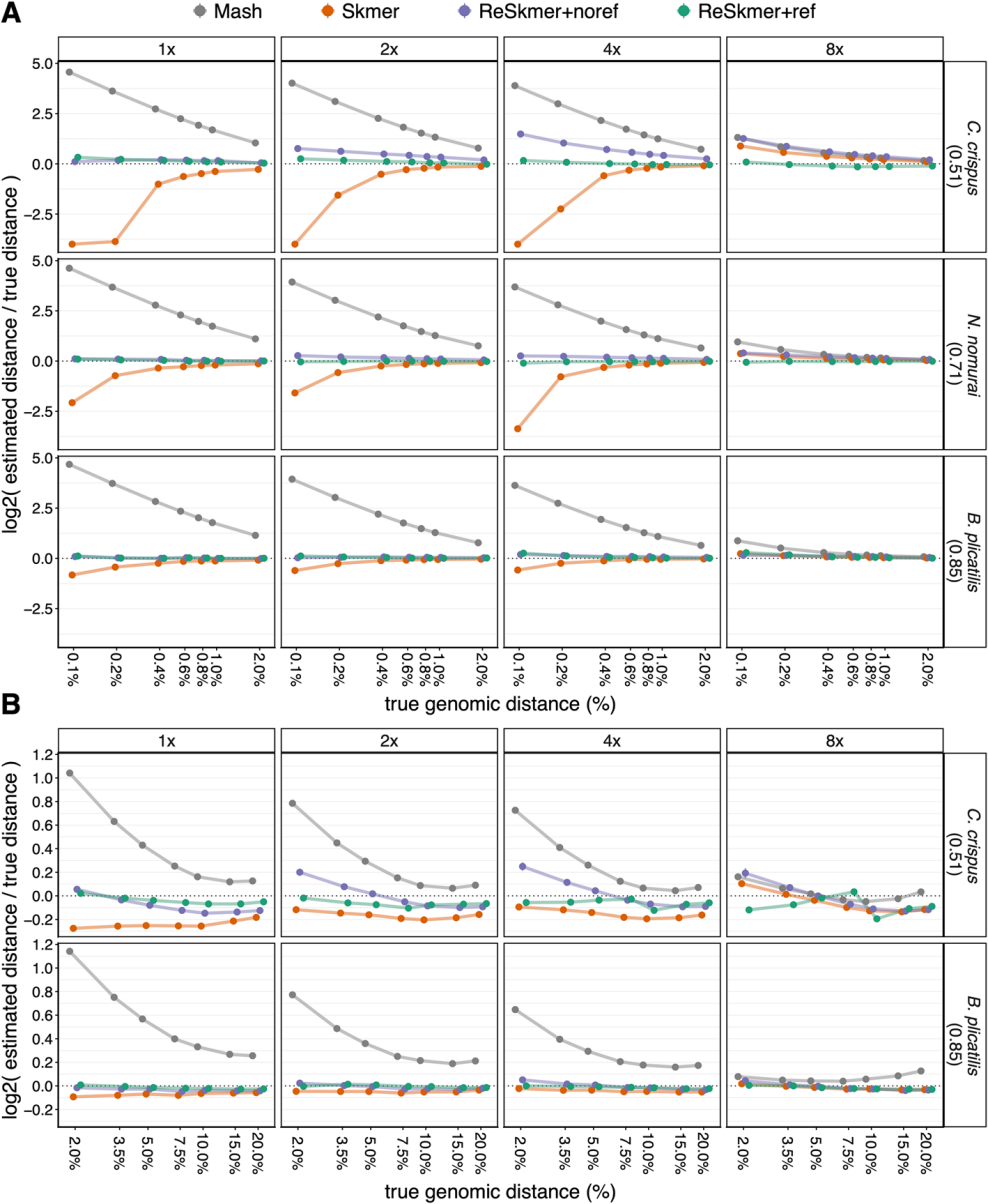
Error in estimated distance estimation on select datasets (see Additional file 1: Fig. S5 for all datasets). **A** Comparing the accuracy of Skmer and ReSkmer in simulated sequencing runs across a range of coverages (top strip) and species (right strip, showing uniqueness ratio parenthetically). The mean log-fold error across replicates, $\log _2(d_{\text {estimated}}/d_{\text {true}})$, (*y*-axis) is plotted against genomic distance (*x*-axis). When $d_{\text {estimated}}=0$, instead of ${-\infty }$, we show $y=-4$ (the red dotted line). **B** Comparison of Skmer methods at higher distances

To isolate the effect of repetitiveness on method accuracy, we first averaged error across all simulated coverages, distances, and replicates. ReSkmer-ref is consistently the most accurate as repetitiveness increases, while Skmer performance notably declines with higher repeats (Additional file 1: Fig. S4). ReSkmer-noref is less accurate than ReSkmer-ref, especially when $\textit{UR}<0.6$, but still remains significantly more accurate than Skmer. Next, to examine the interaction between true distance, uniqueness ratio, and accuracy, we evaluated method performance across individual genomes while varying population genomic distances. Neither coverage-agnostic Mash nor repeat-agnostic Skmer has sufficient resolution to compare repetitive genome skims at the lowest distances, a problem that ReSkmer solves (Fig. 2A, Additional file 1: Fig. S5). Notably, Skmer estimates $d=0$ at the smallest simulated distances for medium to high repeat genomes (UR 0.6–0.4) at low coverages. ReSkmer-ref readily outperforms or matches other methods across all levels of distance, coverage and uniqueness ratios. The improvements are most substantial for smaller distances and more repetitive genomes but are present everywhere. When distances increase to 1% or higher, Skmer starts to become broadly accurate, even for repetitive genomes. However, even there, ReSkmer-ref continues to be more accurate, except for the *S. moellendorffii* and *C. crispus* at $8\times$ coverage (Additional file 1: Fig. S6). Mash dramatically over-estimates distances for coverage below 8$\times$ and never outperforms Skmer or ReSkmer.

ReSkmer-noref also outperforms Skmer in almost all conditions (except in some cases with $4\times$ and $8\times$ coverage), with the most substantial improvements for distances in the 0.1% – 0.5% range for highly repetitive genomes. Not surprisingly, ReSkmer-noref shows higher error compared to ReSkmer-ref, but in many conditions the increase is minimal. In the most extreme case (*C. Crispus* at 0.1% distance and $4\times$ coverage), ReSkmer-noref computes a distance of 0.20% compared to 0.12% with ReSkmer-ref. The slightly higher over-estimation of ReSkmer-noref than ReSkmer-ref is likely due to remaining inaccuracies in RESPECT spectra. Skmer underestimates distances for $4\times$ coverage or less and overestimates for $8\times$ (Skmer changes how it deals with errors for $>4\times$ coverage, as noted earlier).

Finally, we test if ReSkmer remains accurate for phylogenetic level distances (5% – 20%). At high distances, using a single reference genome for *R* can become problematic, because in our simulations, accumulated substitutions only break repeats and increase UR (Additional file 1: Fig. S1). Thus, ReSkmer-ref can have higher error than other methods in some conditions, such as in $8\times$ genome skims of *C. crispus* when $d=10\%$ (Fig. 2B). In spite of these corner cases, ReSkmer-ref is more accurate than both Skmer and ReSkmer-noref in most scenarios. ReSkmer-noref is always at least as accurate as Skmer, and reduces the error substantially for low coverage and highly repetitive genomes. Mash, as expected, is sensitive to coverage: it performs well with $8\times$ but has very high error at $4\times$ or lower. Overall, ReSkmer-noref is preferred at larger (phylogenetic) distances.

#### ReSkmer-noref outperforms ReSkmer-ref with lower quality assemblies

**Fig. 3:**
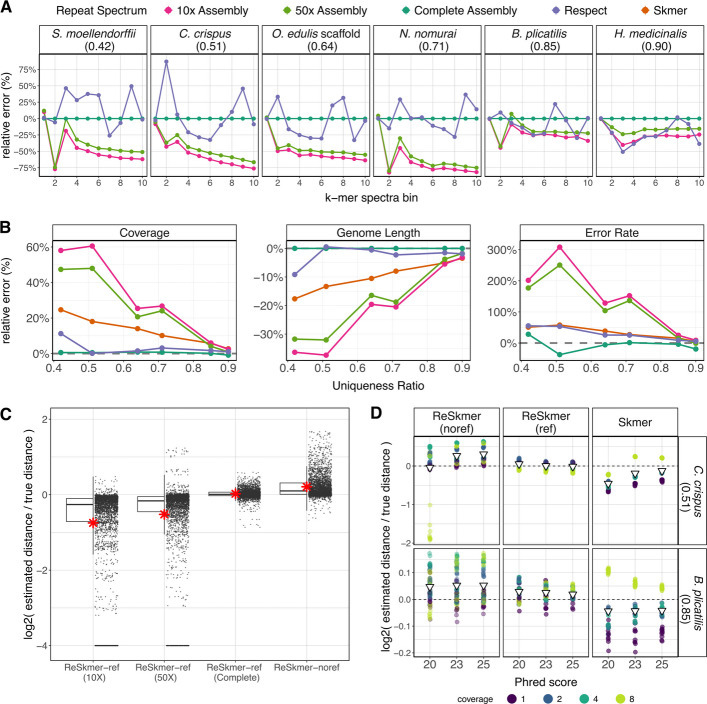
Robustness to parameter estimation. **A** Accuracy of estimated repeat spectra ($\hat{r}_i$) computed using Jellyfish from Megahit assemblies (Illumina reads, $10\times$ and $50\times$ coverages) or using RESPECT from a $4\times$ genome skim. We show ${}^{\hat{r}_i\!}/_{\!r_i}-1$ for $i\le 10$ (*x* axis). “Complete Assembly” refers to the finished genome which provides the reference spectrum ($r_i$). **B** The effect of assembly completeness (color) and repetitiveness (UR on the *x-*axis) on the accuracy of parameter estimation (*y-*axis). **C** The effect of inaccurate repeat spectra on the log-fold error in ReSkmer distance estimation across all genomes, coverages, and distances. Boxplots show the median log-fold error (crossbar) and quartiles for estimates of *d*. On the right, log-fold error of each measure of *d* is plotted. The asterisk represents the mean for each method. **D** Log distance error across Skmer versions with reads simulated at different Phred quality scores, *d*=1%, and all coverages. Triangles represent the mean

Since using repeat spectra estimated from reference genomes shows better accuracy compared to repeat spectra estimated using RESPECT, we next assess if the same remains true with lower-quality assemblies. We simulate Illumina paired-end reads from each genome at $10\times$ and $50\times$, assemble the reads using Megahit [44] and Spades [45], and use the assemblies to compute repeat spectra used for ReSkmer-ref. Megahit assemblies consistently underestimate repetitiveness (e.g., $r_2 \ldots r_{10}$), especially in high-repeat conditions (Fig. 3A). Results are similar for Spades assemblies, except for the lowest repeat condition ($\textit{UR}=0.90$), where $r_{i\ge 4}$ are overestimated (Additional file 1: Fig. S7). RESPECT applied to $4\times$ skims has a mix of over- and underestimation of $r_{i\ge 2}$ values, but its repeat spectra are generally more accurate than low-quality assemblies.

The underestimation of repeat *k*-mers in lower quality assemblies in turn impacts ReSkmer’s ability to estimate sequencing parameters (Fig. 3B). Low-quality assemblies (especially in genomes with low uniqueness ratios) lead to an overestimation of coverage ($61\%$ for the $10\times$ assembly), underestimation of genome length (by as much as $37\%$), and a large overestimation of error rate ($308\%$). As the uniqueness ratio increases, the importance of repeat spectra in estimating other sequencing parameters diminishes, making all estimates similar to each other.

Concurrent with decreased accuracy of sequencing parameter estimation, ReSkmer-ref also tends to underestimate distance when used with short-read assemblies at all coverages (Fig. 3C). Expectedly, $50\times$ sequencing coverage performs better than $10\times$ but remains far from the accuracy of ReSkmer-ref using a finished reference assembly. Distances obtained using Spades assemblies are still underestimated on average, although a small subset of them substantially overestimate distances (Additional file 1: Fig. S8). In contrast, ReSkmer-noref tends to overestimate distance. However, the overestimation by ReSkmer-noref is lower than the underestimation experienced by ReSkmer-ref with either of the tested assemblers. Overall, ReSkmer-ref with short-read assemblies is less accurate than ReSkmer-noref, both in terms of sequencing parameter estimation and genomic distance calculation.

#### Higher error rates are tolerated better by ReSkmer

We next test the robustness to sequencing error rates by simulating reads for *d* = 1% with three Phred scores (20, 23, 25), applied to a highly repetitive (*C. crispus* at UR = 0.51) and a low repeat (*B. plicatilis* at UR = 0.85) genome. All methods estimate error accurately in low-repeat conditions (Additional file 1: Fig. S9). For the high-repeat genomes, Skmer tends to overestimate error by a significant amount ($\approx$ 50%) and particularly fails at the combination of high-error and high-coverage (though at $>4\times$, Skmer does not use $\epsilon$ estimates). ReSkmer-noref also overestimates error, but often less so than Skmer, and does not fail at the high-error/high-coverage condition. ReSkmer-ref is easily the best-performing method with little to no underestimation of the sequencing error rates.

When considering error in distance computation, all methods are robust to error for low-repeat genomes (Fig. 3D; bottom panel). For high-repeat genomes, Skmer is negatively impacted with increased sequencing error $\epsilon$ (top panel). ReSkmer-ref is mostly robust to $\epsilon$, with little change in its estimates as error changes. ReSkmer-noref has higher error than ReSkmer-ref, but nevertheless is mostly robust to error. However, it can underestimate distance when coverage is high (8$\times$), the genome is repetitive, and sequencing error is high (Phred = 20).

### Biological data

We next test if ReSkmer retains its advantage on three published biological datasets (Additional file 1: Table S2). In each case, we perform decontamination and subsample reads to control coverage at $1\times \ldots 6\times$. See Biological data pre-processing section for details. Since the true values of distance are not known, we instead measure nucleotide diversity of populations (hereafter referred to as $\theta$) and focus our evaluations on 1) robustness of the estimates to the coverage, 2) matching of the per-population estimates to estimates from methods that require mapping reads to a reference genome, and 3) separation among populations (when available).

#### Reference-free ReSkmer separates *Apis* populations and stabilizes $\theta$ estimates

We study a Western honey bee (*Apis mellifera*) dataset [46] comprised of three populations (BM, MAS, and FAS), made up of 40 colonies for a total of 120 sequenced samples. The FAS and MAS populations are recently derived from the parent BM population, and have been separated geographically for three generations. DNA was extracted from male drone honey bees, then sequenced with an Illumina HiSeq 2500 machine with a stable read length of approximately 100bp and a mean coverage of $33\times$. To evaluate the similarity of our estimates against mapping-based methods, we run ANGSD [11] on full-coverage data.

**Fig. 4:**
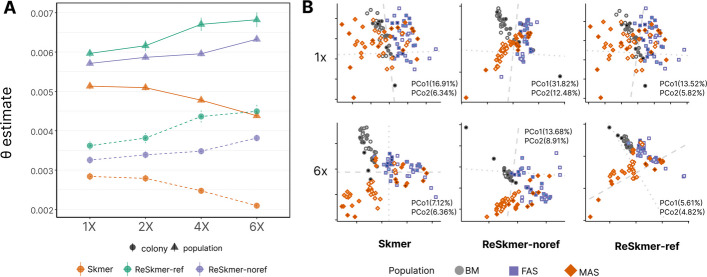
*Apis mellifera* data results: **A** Measurement of $\theta$ (*y*-axis) as estimated by different versions of Skmer across different levels of sequencing depth (*x*-axis). Solid lines with triangles represent measurements of nucleotide diversity of bee populations, while the dashed lines with circles are measurements belonging to colonies. **B** PCo1 and PCo2 of PCoA analysis of distance matrices produced by Skmer. Each of the three honey bee populations is differentiated by color, and members of the same hive have the same shade of their population color. Plots of ReSkmer have been rotated with the procrustes method to align populations with those of Skmer. The direction of the original axes are denoted by a dashed line (PCo1) and dotted line (PCo2)

Expectedly, all methods estimate $\theta$ within colonies – recombinant haploid samples of one queen bee – to be significantly smaller than population-level estimates (Fig. 4A). ReSkmer $\theta$ estimates are higher than Skmer estimates, increasing values by 16–56% in populations and 28–114% in colonies, depending on the coverage. These results are consistent with simulations, where Skmer underestimates distances. The ReSkmer and Skmer estimates of $\theta$ both strongly correlate with ANGSD. Pearson correlation coefficients range from $0.58-0.77$ across coverage values and choice of method (Table 1; Additional file 1: Fig. S10). On this dataset, at the highest coverage does Skmer substantially outperform all other methods ($R=0.90$). However, they all outperform coverage-agnostic Mash at low sequencing depth, where it has low correlation at $1\times$ (R = 0.10) and medium correlation at $2\times$ (R = 0.35) (Table 1; Additional file 1: Fig. S11).

**Table 1:** Correlation table

	*Apis mellifera*				*Drosophila melanogaster*			
	Mash	Skmer	ReSkmer (ref)	ReSkmer (noref)	Mash	Skmer	ReSkmer (ref)	ReSkmer (noref)
$1\times$	0.10	0.63	0.64	**0.65**	0.12	0.58	**0.88**	0.76
$2\times$	0.35	0.71	0.66	**0.76**	0.12	0.61	**0.90**	0.86
$4\times$	0.56	**0.87**	0.63	0.81	0.40	0.69	0.88	**0.90**
$6\times$	0.56	**0.90**	0.61	0.66	0.31	0.40	0.83	**0.90**

Correlation of $\theta$ estimated using *k*-mer-based methods versus mapping to reference genomes (ANGSD at $33\times$ coverage for *Apis mellifera* and Unified Genotyper from original study [47] for *Drosophila melanogaster*). We show the Pearson correlation coefficient. Bolded: the highest correlation per coverage

In terms of robustness to coverage, ReSkmer-noref is the most stable measure of $\theta$ (Fig. 4A) with a change in $\theta$ of 11% and 18% for populations and colonies, respectively, compared to 14% and 26% for Skmer and 15% and 24% for ReSkmer-ref. Mash estimates of $\theta$ are much more sensitive to coverage with a change of 66% in populations and 70% in colonies (Additional file 1: Fig. S12).

Next, we test how well different methods detect population structure. We perform PCoA on distance matrices produced by each method. Plotting PCo1 and PCo2 (Fig. 4B; Additional file 1: Fig. S13) shows that only ReSkmer-noref separates the three populations with little overlap at all coverages. While at $6\times$ all Skmer-based methods discriminate populations clearly, at low coverages (1$\times$ – 4$\times$) both Skmer and ReSkmer-ref fail to separate the “MAS” population. Mash also does not differentiate populations in $1\times$ and $4\times$ data (Additional file 1: Fig. S14). ANGSD clustering at $4\times$ is comparable to ReSkmer-noref at all coverages, suggesting any remaining observed overlaps may be due to real genomic similarity.

#### ReSkmer correlates with published measures of $\theta$ in *Drosophila melanogaster*

We obtained a dataset from the *Drosophila* Genome Nexus [47] comprised of 21 different populations sequenced with Illumina Genome Analyzer IIx. In this dataset, populations have different numbers of samples, sequencing libraries, and average read lengths. Skmer estimates of $\theta$ are compared with published values obtained using a high-coverage analysis using Unified Genotyper [48].

**Fig. 5:**
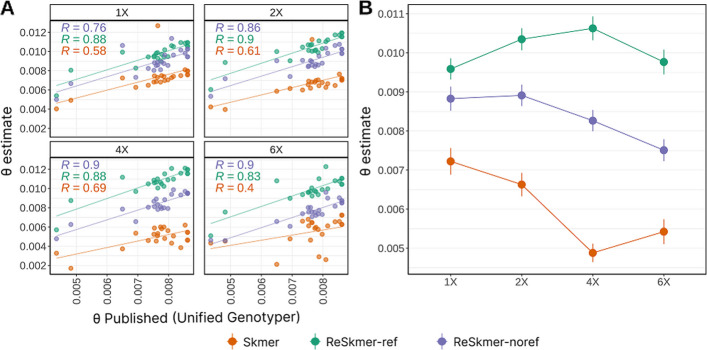
*Drosophila* Genome Nexus results. **A** Correlation plots of different Skmer estimates of $\theta$ compared to published estimates with a GATK analysis. **B** Mean measure of $\theta$ (*y*-axis) across all *Drosophila* populations as estimated by different versions of Skmer across different levels of sequencing depth (*x*-axis)

Both ReSkmer versions readily outperform Skmer and Mash across all coverages in terms of correlation with published Unified Genotyper estimates of $\theta$ (Table 1; Fig. 5A). Notably, Skmer performs worse at the highest coverage ($6\times$) with Pearson correlation dropping to $R=0.40$. Mash has a similar trend, with correlation peaking in $4\times$ data ($R = 0.40$), then decreasing in $6\times$ data ($R = 0.31$) (Additional file 1: Fig. S15). Skmer also greatly overestimates the nucleotide diversity of one population (“CO”) at 1–2X, but is able to correct its measurement at higher coverages (Additional file 1: Fig. S16). Both ReSkmer versions measure reasonable estimates for this population.

Both versions of ReSkmer are also more stable than Skmer across coverages. ReSkmer-ref and ReSkmer-noref estimates of $\theta$ change by $11\%$ and $16\%$ respectively, compared to Skmer which changes $23\%$ (Fig. 5B). Mash is highly unstable (Additional file 1: Fig. S17) and produces unreasonable distances for population-level samples ($2\%< \theta < 4\%$). ReSkmer again estimates larger values of θ when compared to Skmer, mirroring both the simulation and honey bee datasets.

#### ReSkmer-noref closely matches published $\theta$ values for diploid organisms

We next ask whether ReSkmer can perform well on a more diverse set of species, including diploid genomes, noting that the ReSkmer modeling does not account for within-sample heterozygosity. We analyze a diverse dataset of diploid samples with 13 populations from 7 different species. We use previously published $\theta$ estimates for each population [49, 50] and compare them against alignment-free estimators. We note that the available assemblies for most of the species analyzed here are of low quality, making ReSkmer-noref the preferred method.

**Fig. 6:**
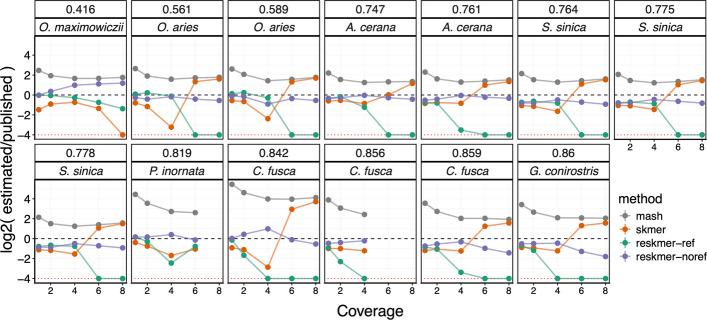
ReSkmer performance on diploid data. Log-fold change between $\theta$ estimated by different alignment-free methods (y-axis) across all diploid species and populations at varying coverages (x-axis). Top strip illustrates the estimated uniqueness ratio for each population using RESPECT at $4\times$ coverage. Seven different species are represented here, *O. maximowiczii* (plant), *O. aries* (mammal), *A. cerana* (insect), *S. sinica* (bony fish), *P. inornata*, *C. fusca*, *G. conirostris* (birds). Some coverage levels are omitted due to low coverage in original analysis. When estimated $\theta=0$, instead of $-\infty$ we show $y=-4$ (dotted red line)

On average, ReSkmer-noref consistently produces the best estimates compared to previously published $\theta$ (Fig. 6). Across all coverages, it has the least mean absolute error ($34\%$) when compared to other methods (ReSkmer-ref: $61\%$, Skmer: $101\%$, Mash: $524\%$). It also has the most stable estimates across coverages, having a small error range of only [$25-49\%$] compared to Skmer’s wide range of error [$46-268\%$]. ReSkmer-ref has very low error at low coverages ($1\times =27\%$ and $2\times =37\%$) in spite of using low quality assemblies in most scenarios, sometimes even outperforming ReSkmer-noref [see *P. inornata* ($\textit{UR}=0.819$) and *C. fusca* ($\textit{UR}=0.842$)]. ReSkmer-ref shows a substantial performance decline at around $4\times$ coverage, where poor-quality genomes lead to distance underestimation, including $\theta =0$ estimates in many cases. Skmer accuracy is erratic across datasets, achieving peak accuracy at around $1\times$ ($46\%$ error). As expected, Mash overestimates $\theta$ at low coverages, however its estimates improve only slightly at higher coverages.

## Discussion

A central theme of this research is that low-coverage whole-genome shotgun sequencing (i.e., genome skimming) can rapidly, without assembly and alignment, provide population genetic information even when a high-quality reference genome is not available. Previous methods for analyzing low-coverage data (e.g., ANGSD) require a reference assembly and map reads to the reference to identify polymorphism and site frequency spectra. Our work shows that neither the use of a reference assembly nor the computationally expensive alignment step is strictly necessary. A collection of sampled *k*-mers from a genome has been known to be sufficient to estimate sequencing parameters for non-repetitive genomes [51]. Similarly, the intersection of *k*-mers sampled from two genomes has been used to estimate the genetic distance (and in turn nucleotide diversity) between two genomes [23, 26]. However, real genomes are quite complex; they may exhibit polyploidy, are repetitive, and therefore, all *k*-mers are not sampled with equal probability. Furthermore, for highly repeated *k*-mers, sequencing errors can create false intersections between *k*-mer sets, changing distance estimates. Our theoretical results and the ReSkmer tool address these limitations and enable estimating small genomic distances between highly repetitive genome skims.

ReSkmer demonstrates robust accuracy across a wide range of taxonomic groups and repetitiveness. The relationship between repetitiveness and error is not readily apparent in the diploid biological datasets (Fig. 6), though Skmer does show a performance decline in our most repetitive empirical dataset (*O. maximowiczii*). The lack of a clear pattern is likely due to confounding variation in other parameters (e.g., true $\theta$, sequencing error rate, genome length, contamination, and ploidy) that may obscure underlying trends. Controlling for these parameters via simulations allows for the impact of repeats to be clearly observed (Additional file 1: Fig. S4). While designed for repetitive Eukaryotic genomes, a quick application of ReSkmer on a less repetitive ($\textit{UR}\approx 0.90$) bacterial dataset [52] shows that it can separate sequence sub-types well regardless of the coverage (Additional file 1: Fig. S19).

On real data, exact values of $\theta$ are difficult to ascertain, as even high-coverage methods run into problems like pseudo-heterozygosity and low-quality mappings [47], and these issues are exacerbated when coverage is low [12, 53]. In many applications, the focus has been on relative values of $\theta$. This is especially true in the biodiversity monitoring context, where what matters is temporal and spatial change in $\theta$. Therefore, in this study, we focused on the *correlation* of our estimates of $\theta$ to those produced by mapping-based methods. Across our simulation and biological analyses, ReSkmer was more accurate and more robust to noisier data (especially at the lowest coverages). Beyond improved overall accuracy and correlation compared to Mash and Skmer, ReSkmer also leads to fewer outliers that are unusually distant from other samples (see an example in Additional file 1: Fig. S16). This is also true in simulations, where both ReSkmer parameter (Additional file 1: Fig. S9) and distance estimation (Fig. 3D) were more robust to noiser, high-error data. Similarly, ReSkmer avoids estimating 0% distance for closely related species, a problem that Skmer can suffer from (Additional file 1: Fig. S5). This improved accuracy did not come at the expense of higher running times (Additional file 1: Table S3); each sample takes less than 5 minutes to analyze. ReSkmer needs a repeat spectrum as input, which can be obtained from an assembly or in a reference-free fashion using RESPECT. While ReSkmer-ref was slightly more accurate than ReSkmer-noref with perfect assemblies used in simulations, patterns changed with less accurate assemblies (Fig. 3). Obtaining a high-quality reference assembly that accurately captures the repeat spectra of a genome may not be feasible with short-read sequencing and will most likely require a combination of short and long-read sequencing in many cases [54, 55]. Additionally, if individuals of the same species show non-trivial differences in repeat structure or genome length, a single assembly may not capture the heterogeneity of a dataset; RESPECT can capture such differences by estimating the spectra per sample. Thus, for many organisms, it may be best to simply estimate the spectrum from the skim instead of relying on incomplete assemblies.

The impact of coverage on the accuracy was not always as expected. On simulated datasets, ReSkmer has higher error when coverage increases to 8$\times$ for some but not all datasets, especially when used with RESPECT (Additional file 1: Fig. S5). On biological data, too, the correlation of ReSkmer-ref estimates with mapping-based estimates was at its highest with $2\times$ coverage; similarly, ReSkmer-noref did not improve above $4\times$ for *Apis mellifera* (Table 1). The reason behind this surprising pattern is not fully clear, but we speculate it could be the result of erroneous *k*-mers becoming an increasing proportion of the unique *k*-mer set as coverage increases. In fact, if we aim to balance the precision of the *k*-mer set (i.e., portion of observed *k*-mers that are error-free) versus the recall (i.e., portion of genomic *k*-mers that are observed), simple theory suggests that 2–4$\times$ coverage is optimal (Additional file 1: Fig. S20). Other reasons for performance decline at higher coverages may be dataset dependent and stem from errors that are neither modeled by ReSkmer nor RESPECT (e.g., contamination, insertions, or PCR bias). As coverage increases, failure to correct these issues may exacerbate their impact. While future algorithmic work should investigate how the accuracy of ReSkmer can improve for high coverage, in the meantime, the result is encouraging in terms of the applications. Results imply that ecological studies can decrease cost by eliminating not just the need for a reference genome but also by sequencing at coverage as low as 1$\times$.

In spite of these advancements, some questions surrounding ReSkmer remain. For example, non-target *k-*mers (contaminants) can impact genomic distance estimation using Skmer [56]. Although we follow a decontamination procedure based on taxonomic read identification in our biological analyses [57], more experiments should be done to characterize the effects of missing contaminants or overzealous read removal on ReSkmer’s accuracy. Secondly, ReSkmer equations are based on the parent-child model we describe here. However, developing more nuanced models of repeat creation and loss may allow us to have a deeper understanding of evolution’s influence on intersections of *k*-mer sets, leading to better estimates of genomic distance. Finally, both Skmer and ReSkmer assume a haploid genome. While our results on diploid genomes were mostly positive, future work can model the effects of sequencing a mixture of multiple chromosomes (i.e. polyploidy) on the intersection of *k*-mer sets, and how that may influence Skmer and ReSkmer accuracy. Despite these limitations, we believe that ReSkmer provides important new algorithmic ideas to allow population genetic analyses of ecological samples using low-coverage, inexpensive genome sequencing for non-model organisms.

## Conclusions

In summary, our findings show that modeling the impact of repeats can increase the accuracy and robustness of alignment-free *k*-mer-based distance estimation methods. In both simulated and biological data, ReSkmer was able to improve upon repeat-agnostic methods (e.g., Mash and Skmer). We were also able to push the boundaries of accurate low-coverage population genomic analyses and produce results that are highly correlated with output from mapping-based methods. Consequently, ReSkmer has the potential to provide a novel approach to accessible and affordable biodiversity monitoring.

## Methods

### ReSkmer algorithm: overview

Several parameters, listed in Table 2 (see also Additional file 1: Section A.1), describe the composition of the genomes and their genome skims; we use a superscript to denote the genome where necessary. We start with an input of two genome skims, each obtained from a single haploid individual, and some (‘known’) parameters which can be directly estimated from the skims. We seek to compute the Hamming distance *d* (if aligned perfectly) between underlying genomes, $G^{(1)}$ and $G^{(2)}$ using *k*-mers alone. We assume all *k*-mers mutate independently, and all locations are sampled without bias. Considering the *k*-mer set of both skims $\mathcal {S}^{(1)},\mathcal {S}^{(2)}$, let $I=\mathcal {S}^{(1)}\cap \mathcal {S}^{(2)}$ denote the intersection set. Instead of computing *I* directly, we approximate it using Mash [26] by computing the Jaccard index and using $|I| = {}^{J}/_{\!(1 + J)}(|\mathcal {S}^{(1)}| + |\mathcal {S}^{(2)}|)$.

**Table 2:** Parameters used in ReSkmer

**Known Parameters**	
*k*	Size of the oligomers used for analysis
B	number of nucleotides sequenced in a genome-skim
$\ell$	mean length of read. Each read contributes $\ell -k+1$ k-mers.
*O*	Observed spectrum $O=[o_1,o_2,\ldots ],$ where $o_h$ is the number of *k*-mers that are sampled *h* times.
$\mathcal {S}$	Set of *k*-mers in a genome-skim. $|\mathcal {S}|=\sum \nolimits _i o_i$.
**Assumed to be known in ancestral genome or estimated using RESPECT** [29]	
$\mathcal {R}$	$\mathcal {R}=[\mathcal {R}_1,\mathcal {R}_2,\ldots \mathcal {R}_m]$, where $\mathcal {R}_i$ is the set of *distinct* *k*-mers that appear in exactly *i* locations.
*R*	Repeat spectrum $R=[r_1,r_2,\ldots ,r_m]$, where $r_i=|\mathcal {R}_i|$
L	Length of genome; $L=\sum \nolimits _i i r_i$.
*c*	Sequence depth; $c={}^{B\!}/_{\!L}$
$\lambda$	*k*-mer coverage= $\mathbb {E}[\text {number of samplings of a k-mer at a specific location}] = c (\ell -k-1)/\ell$
**Unknown Parameters**	
$\epsilon$	Sequencing error= $\Pr [\text {an arbitrary nucleotide is read erroneously in a read}]$
$\xi$	$\mathbb {E}[\text {number of error-free samplings of a k-mer at a specific location}] = \lambda (1-\epsilon )^k$
$\eta$	$\Pr [\text {an arbitrary k-mer at a specific location is sampled without error}]=1 - e^{-\lambda (1 - \epsilon ) ^ k}$
$\zeta$	$\mathbb {E}[\text {the total number of k-mers generated from a position i}] = \eta + \lambda (1 - (1 - \epsilon ) ^ k)$

In order to estimate *d*, we derived (see Modeling the number of shared *k*-mers) an estimate of the expected size of the intersection set $\vert \mathbb {E}[|I|]\vert$ as a function of *d* and several sequencing parameters

$$\begin{aligned} \mathbb {E}[|I|]=\mathcal {I}\left( d;{R}^{(1)},\lambda ^{(1)},\epsilon ^{(1)},\eta ^{(1)},\lambda ^{(2)},\epsilon ^{(2)},\eta ^{(2)}) \right) . \end{aligned}$$

Notably, this derivation assumes that genome $G^{(1)}$ is the parent of $G^{(2)}$, and each position of $G^{(1)}$ is mutated under the JC69 model with probability $- {}^{3\!}/_{\!4}\ln (1-{}^{4\!}/_{\!3}d)$ to obtain $G^{(2)}$. It only requires knowledge of ${R}^{(1)}$, and provides an asymmetric estimate of the distance as

5 $$\begin{aligned} d_1^* = \arg \underset{d}{\min }\ \left\lvert |I|- \mathcal {I}(d;{R}^{(1)},\lambda ^{(1)},\epsilon ^{(1)},\eta ^{(1)},\lambda ^{(2)},\epsilon ^{(2)},\eta ^{(2)}) \right\rvert \;. \end{aligned}$$

In practice, we perform calculations both ways for a pair of genomes.

#### Optimization for *d*

We solve this optimization problem of Eqn. 5 using the hyperbolic Brent method [43] for finding roots, implemented in Scipy, bounded to $d\in [0,1]$. The Brent method requires that the function (here, $I-\mathcal {I}(d)$) changes sign in the bounds given ($d\in [0,1]$). It can be easily checked that $\mathcal {I}(1)=0$, and since $I\ge 0$, we only need that $I\le \mathcal {I}(0)$. If the model is absolutely correct, all parameters are calculated correctly, and observations match the expectations, this condition will be satisfied. When *d* is small, it is possible to observe intersection sizes that are larger than what one would expect even with $d=0$. For example, if the distance is close to zero and the error is overestimated, we may get $I> \mathcal {I}(0)$. In such conditions, the Brent method will fail, and we return a 0% distance. Finally, since Eq. (5) is asymmetric, ReSkmer outputs two distances for any pair. The complete procedure is described in Algorithm 1.

**Figure:**
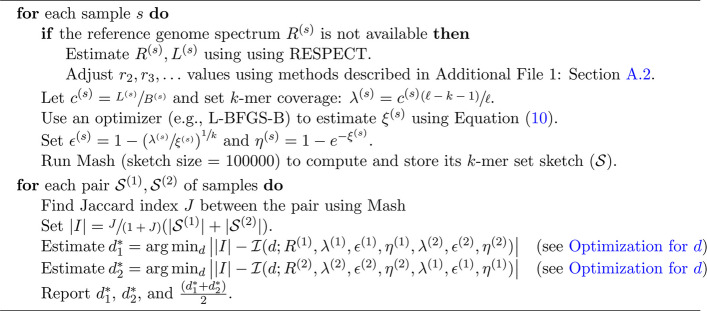
**Algorithm 1** ReSkmer

### Modeling the number of shared *k*-mers

In this section, we derive an estimate of the size of the intersection given by Eq. (3), and reproduced below

$$\begin{aligned} \mathbb {E}&[|I|] = \mathcal {I} (d;{R}^{(1)},\lambda ^{(1)},\epsilon ^{(1)},\eta ^{(1)},\lambda ^{(2)},\epsilon ^{(2)},\eta ^{(2)}) \approx \\&\sum \limits _{i=1}^{m} r_i^{(1)} \left( 1-\left( 1-\eta {^{(1)}}\right) ^i \right) \left( 1-\left( 1-\eta ^{(2)}(1-d)^k\right) ^i\right) + 3k r_i^{(1)} \left( 1- e^{- i b \lambda ^{(1)} \epsilon ^{(1)} (1-\epsilon ^{(1)})^{k-1}}\right) \\&\quad \left( 1 - \left( (1-d)^k e^{ - b \lambda ^{(2)} \epsilon ^{(2)} (1-\epsilon ^{(2)})^{k-1}} - bd(1-d)^{k-1}\eta ^{(2)}+ \left( 1- (1-d)^k \right) \right) ^{i}\right) \end{aligned}$$

where $b= k (e^{-{}^{1\!}/_{\!3k}}-1)\approx {}^{-1\!}/_{\!3}$ is a constant.

To model the impact of repeats on the intersection of genome skims, we partition *I* into two components: (a) $I_0$ denotes the set of *k*-mers that are sampled without error in skim 1 and skim 2 and are not mutated in the evolution from parent to child, and (b) $I_1$ denotes the set of *k*-mers that are mutated exactly once in $G^{(2)}$, but still match a *k*-mer in skim 1. Note that $|I| \ge |I_0|+|I_1|$, since *k*-mers can be mutated multiple times and still match up in the intersection. However, the probability of such events is small enough that we can ignore them and use $|I| \approx |I_0|+|I_1|$, and thus, $\mathbb {E}[|I|] = \mathbb {E}[|I_0|]+\mathbb {E}[|I_1|]$.

#### Error-free intersection ($\vert I_0\vert$).

Consider a *k*-mer $u\in \mathcal {R}_i$. Let $P_0^{(1)}$ be the probability that at least one copy of *u* is sampled without error in $\mathcal {S}^{(1)}$. Clearly, $P_0^{(1)} = 1-\left( 1-\eta ^{(1)}\right) ^i$ because, by definition, each of the *i* copies has probability $\eta ^{(1)}$ of the event.

Similarly, let $P_0^{(2)}$ be the probability that at least one copy of *u* is unmutated and sampled without error in $\mathcal {S}^{(2)}$. Since sampling and mutations are independent, we multiply $(1-d)^k$ and $\eta ^{(2)}$ to get $P_0^{(2)} = 1-\left( 1-\eta ^{(2)}(1-d)^k\right) ^i$. Since the sampling in both genomes is independent, $\Pr [u\in I_0] = P_0^{(1)}P_0^{(2)}$. Summing over all *k*-mers,

6 $$\begin{aligned} \mathbb {E}[|I_0|] = \sum \limits _{i=1}^m r_i^{(1)} P_0^{(1)}P_0^{(2)} = \sum \limits _{i=1}^m r_i^{(1)} \left( 1-\left( 1-\eta ^{(1)}\right) ^i \right) \left( 1-\left( 1-\eta ^{(2)}(1-d)^k\right) ^i\right) \end{aligned}$$

#### Intersection due to sequencing error ($|I_1|$).

Any *k*-mer *u* has exactly 3*k* neighbors at Hamming distance 1 (i.e., ‘1-neighbors’). Consider two lists containing $n_1$ and $n_2$ randomly picked 1-neighbors of *u*, with replacement. For any 1-neighbor, the probability that it is in both lists is

$$\begin{aligned} \left( 1- \left( 1-\frac{1}{3k}\right) ^{n_1}\right) \left( 1-\left( 1-\frac{1}{3k}\right) ^{n_2}\right) \approx \left( 1-e^{-\frac{n_1}{3k}} \right) \left( 1-e^{-\frac{n_2}{3k}} \right) \end{aligned}$$

and, thus, the expected size of the intersection of the two lists is approximately

$$\begin{aligned} 3k \left( 1-e^{-\frac{n_1}{3k}} \right) \left( 1-e^{-\frac{n_2}{3k}} \right) \;. \end{aligned}$$

In our setting, 1-neighbors of *k*-mers are generated due to either mutations in the second genome or errors during sequencing; thus, $n_1,n_2$ are random quantities, which we denote by $N^{(1)}, N^{(2)}$. Since the processes are independent for the two genome skims, $\mathbb {E}[ \left( 1-e^{-{}^{N^{(1)}\!}/_{\!3k}} \right) \left( 1-e^{-{}^{N^{(2)}\!}/_{\!3k}} \right) ] = \mathbb {E} [1-e^{-{}^{N^{(1)}\!}/_{\!3k}}] \mathbb {E}[1-e^{-{}^{N^{(2)}\!}/_{\!3k}} ] = \left( 1-\mathbb {E} [e^{-{}^{N^{(1)}\!}/_{\!3k}}]\right) \left( 1-\mathbb {E}[e^{-{}^{N^{(2)}\!}/_{\!3k}} ]\right)$. For a *k*-mer repeated *i* times in $G^{(1)}$, we can write $N^{(1)} = \sum \nolimits _{j=1}^i M^{(1)}_{j}$ where $M^{(1)}_{j}$s are the number of 1-neighbors for each copy and are *i.i.d*. Similarly, for $N^{(2)}$, we can write it as a sum of *i.i.d* r.v.s (groupings are based on the orthologous copy in $G^{(1)}$). Due to the independence of *k*-mer copies, $\mathbb {E}[e^{-{}^{N^{(1)}\!}/_{\!3k}} ]=\mathbb {E}[e^{-{}^{M^{(1)}\!}/_{\!3k}} ]^i$ and ditto for $N^{(2)}$. Moreover, we have $r_i^{(1)}$ *k*-mers repeated *i* times in $G^{(1)}$, and thus,

7 $$\begin{aligned} \mathbb {E}[|I_1|] = \sum \limits _{i=1}^{m} r_i^{(1)} 3k \left( 1-\mathbb {E} [e^{-\frac{M^{(1)}}{3k}}]^i\right) \left( 1-\mathbb {E}[e^{-\frac{M^{(2)}}{3k}} ]^i\right) \;. \end{aligned}$$

Let $C\sim \text {Poisson}(\lambda ^{(1)})$ be the coverage of *each* copy in the first skim and $X_{jh}$ be an indicator variable showing if sample *h* of a copy *j* has a single error. Clearly, $M^{(1)}_j = \sum \nolimits _j^{C} X_{jh}$ and *X*s are *i.i.d*. Letting $\alpha _1$ be the shorthand for $P(X) = k \epsilon ^{(1)} (1-\epsilon ^{(1)})^{k-1}$, we can arrive at $\mathbb {E} [e^{-{}^{M^{(1)}\!}/_{\!3k}}] = \mathbb {E} [\mathbb {E} [e^{-{}^{M^{(1)}\!}/_{\!3k}};C]] = \mathbb {E} [\mathbb {E} [(e^{-{}^{1\!}/_{\!3k}})^{X}]^{C}]$. Recalling the probability generating function (PGF) of an indicator variable ($\mathbb {E}[z^x]= p z + 1- p$), we get $\mathbb {E} [(e^{-{}^{1\!}/_{\!3k}})^{X}]= \alpha _1 e^{-{}^{1\!}/_{\!3k}} + 1- \alpha _1$.

Using the PGF of the Poisson distribution ($e^{\lambda (z -1)}$), and defining $b= k (e^{-{}^{1\!}/_{\!3k}}-1)\approx {}^{-1\!}/_{\!3}$ as a shorthand, we get

8 $$\begin{aligned} \mathbb {E} [e^{-\frac{M^{(1)}}{3k}}] = \mathbb {E} [\left( \alpha _1 e^{-\frac{1}{3k}} + 1- \alpha _1\right) ^{C}] =e^{\lambda ^{(1)} \left( \alpha _1 e^{-\frac{1}{3k}} + 1- \alpha _1 - 1\right) } = e^{\lambda ^{(1)} \epsilon ^{(1)} (1-\epsilon ^{(1)})^{k-1} b} \; \end{aligned}$$

For $\mathbb {E} [e^{-{}^{M^{(2)}\!}/_{\!3k}}]$, we need to model both 1-neighbors generated through a mutation and no errors or no mutation and an error. We work through these two cases in Additional file 1: Section A.3 to arrive at

9 $$\begin{aligned} \mathbb {E} [e^{-{}^{M^{(2)}\!}/_{\!3k}}] = (1-d)^k e^{ - b \lambda {^{(2)}} \epsilon (1-\epsilon )^{k-1}} - b d (1-d)^{k-1} \eta ^{(2)} + \left( 1- (1-d)^k \right) \; . \end{aligned}$$

#### Final equation.

Combining $\mathbb {E}[|I_0|]+\mathbb {E}[|I_1|]$ and Eqs. (6) to (9), we get Eq. (3).

### Estimating genomic parameters

We consider *k*-mers in a genome of length *L* and assume that $k\gg \log _4 L$ so that any *k*-mer is unlikely to appear more than once, unless it is part of a repeated sequence. Below, we describe how each needed parameter is calculated.

#### Estimating $r_i$ and $\lambda$.

We assume the repeat spectrum of the genome is already known. If a reference genome is available, this spectrum can be easily calculated. Otherwise, we use RESPECT [29] to estimate the spectrum; RESPECT infers *R* from *O* using a constrained optimization problem and is designed to handle low coverage. Additionally, we use a procedure described in Additional file 1: Section A.2 to adjust $r_i$ and to smooth the output of RESPECT in log space. Given repeat spectrum, we can easily compute base pair coverage $c= {}^{\sum \nolimits _i r_i\!}/_{\!B}$ and set *k*-mer coverage: $\lambda \ = c(1-{}^{(k-1)\!}/_{\!\ell })$.

#### Estimating $\epsilon$.

Assuming each base pair is sequenced erroneously with probability $\epsilon$, let $\xi$ denote the expected *error-free k-mer coverage*, and note $\xi = \lambda (1-\epsilon )^k$. We estimate $\hat{\xi }$ and set $\epsilon = 1-\left( {}^{\hat{\xi }\!}/_{\!\lambda }\right) ^{1/k}$. The number of times a unique *k*-mer repeated *i* times in the genome is sampled without an error follows a Poisson distribution with rate $i \xi$. Denote $o_h$ as a sample allocation to random variable $O_h$, whose expected value, $\mathbb {E}[{O_h}]$, depends upon the repeat spectrum $\textbf{r}$, $\lambda$, and $\epsilon$. By assumption, $\mathbb {E}[{O_h}] = \sum \nolimits _{i\ge 1} r_i f_P(h;i\xi ) + \varepsilon _h,$ where $\varepsilon _h$ is the expected number of k-mers that appear *h* times due to errors and $f_P(h)$ is the PMF of the Poisson distribution. Unfortunately, $\varepsilon _h$ is hard to model. However, we do know that, due to the random nature of errors, it decays rapidly, and for larger *h*, we can set $\varepsilon _h=0$. Thus, for a carefully selected range $H_1\le h \le H_2$, we can write: $o_h \approx \sum \nolimits _{i = 1}^{m} r_i f_P(h;i\xi )$. We then estimate $\xi$ using a weighted least-square estimator:

10 $$\begin{aligned} \hat{\xi } = \arg \underset{\xi }{\min }\ \sum \limits _{h=H_1}^{H_2} \frac{1}{{o_h}}\left( o_h - \sum \limits _{i = 1}^{m} r_i \left( \frac{e^{-i\xi }(i\xi )^h}{h!} \right) \right) ^2 \end{aligned}$$

where the ${}^{1\!}/_{\!o_{h}}$ weights are meant to prevent the estimation from being dominated by the larger $o_h$ values. For $H_1$, to avoid bins that include erroneous *k*-mers, we set it to $\lfloor \lambda \rfloor$ or 2, whichever is larger. For $H_2$, we empirically detected that the choice can be consequential. Thus, instead of using a fixed value, we explore a range of values, and choose the $H_2$ value that results in the lowest mean least square error (see Additional file 1: Section A.4). We solve the optimization problem using the L-BFGS-B algorithm [58] as implemented in Scipy, within bounds $[\lambda (1-0.03)^k,\lambda (1-0.0001)^k]$, corresponding to the $\epsilon \in [0.0001,0.03]$ range (see Additional file 1: Section A.4 for justification of the bounds).

### Experimental procedures

#### ReSkmer data processing

Following the parent/child model, our equations are asymmetric with respect to the two skims. As a result, ReSkmer outputs an asymmetric distance matrix. However, since real data do not have a parent and child, but rather, two contemporary samples, here, we compute and report the average of the two calculated distances $d(G^{(1)}, G^{(2)} | R^{(1)})$ and $d(G^{(2)}, G^{(1)} | R^{(2)})$.

The input spectrum *R* depends on the mode. In the ReSkmer-ref version, we use the repeat spectrum of the reference genome in both comparisons. In the reference-free (ReSkmer-noref) version, we use RESPECT to estimate both $R^{(1)}$ and $R^{(2)}$. RESPECT reaches peak accuracy at $4\times$ coverage (Additional file 1: Fig. S18). Therefore, the use of any RESPECT-estimated spectrum of skims above that threshold will lead to more inaccurate distances. Here we use a protocol that downsamples high-coverage skims to $4\times$, then uses estimated spectra based on those skims as ReSkmer input for higher-coverage distance estimation.

#### Simulation procedures

For each of these assemblies serving as a parent genome $\mathcal {S}^{(1)}$, we simulate an array of child genomes $\mathcal {S}^{(2)}$ with population genetic level genomic distances (0.1% – 2%) by randomly introducing SNPs. Clearly, these simulations do not capture all types of mutations. For example, repetitive elements arise through diverse biological mechanisms (e.g., retrotransposons and gene duplication), whereas in our simulations, repeats are present at the parent and can only be broken with substitutions, introducing no new repeats. In addition, contamination commonly present in biological data is not modeled here and may further influence performance. Although these simulations do not capture all of the complexities of biological datasets, they do provide a controlled framework for assessing method performance.

We use ART [59] to simulate Illumina short read sequencing runs (Phred score of 25, $\ell = 150$) at four coverage levels ($c\in \{1, 2, 4, 8\}$) for each of the parent and child genomes, replicating the process 10 times. For one repetitive genome (*C. crispus*) and one non-repetitive genome (*B. plicatilis*) we generate 10 more replicates of child genomes simulating phylogenetic distances (5% – 20%) and simulate Illumina sequencing using the same parameters. For these two genomes, we take the parent genome and a child genome ($d = 1\%$) and generate skims with two higher sequencing error rates (phred scores 20 and 23) at the same four coverage levels. Finally, to simulate low quality assemblies, we also use ART to generate high-coverage ($10\times$ and $50\times$) Illumina paired-end sequencing (phred score of 25, $\ell = 150$) from our set of assemblies. Then we reassemble them using Megahit.

#### Biological data pre-processing

Due to the potential impacts of contamination [60], we first remove reads that appear to belong to bacteria, archaea, or humans. We utilize a published protocol [61], which includes the removal of adapters and deduplication of reads using BBtools [62], bacterial and archaeal read removal using CONSULT-II [63], and human read removal using Kraken2 [64]. To test the method with low-coverage skims, we use the reference assembly to obtain an initial genome size, then subsample the coverage of all genome skims to target coverages (1$\times$, 2$\times$, 4$\times$, and 6$\times$).

For running ReSkmer-ref, we used available reference genomes: RefSeq GCF_003254395.2 for bees and GCF_000001215.4 for *Drosophila*. For the *Drosophila assembly*, we removed the Y chromosome (all analyzed samples are haploid embryos) and all unmapped contigs. Here we analyze a subset of samples belonging to flies obtained from the Langley and Pool labs.

To assess population genetics using mapping-based methods in the *Apis mellifera* dataset, we calculated $\theta$ based on the Site Frequency Spectrum (SFS) for the three colonies. We used ANGSD v0.940 [11] to compute the unfolded global estimate of the SFS and extract the nucleotide. ANGSD was run under -doSaf 1 to estimate the site allele frequency likelihood for each bee colony/hive. To account for the haploid genome of the target species, we added the -isHap 1 flag.

To assess the accuracy of $\theta$ across species with varying levels of repetitiveness, we analyzed one plant population (*O. maximowiczii*) from a previously published dataset [50], along with populations from a second dataset (SPrUCE) [49]: two mammal populations (*O. aries* [65]), two insect populations (*A. cerana* [66]), three fish populations (*S. sinica* [67]), and five bird populations (*P. inornata*, *C. fusca*, *G. conirostris* [68]). For ReSkmer-ref, we used the same reference genomes employed in the original analyses: GCA_051622875.1 for *O. maximowiczii* and Megahit assemblies from the SPrUCE dataset.

## Supplementary Information

Additional file 1: Supplementary Tables S1-S3, Supplementary Figures S1-21, and Supplementary Notes A1-A4 containing mathematical derivations and algorithmic details [71].

## Data Availability

The code for ReSkmer [69] is available at https://github.com/echarvel3/ReSkmer and published in Zenodo under a CC BY 4.0 license. Scripts [70] used to simulate data and results for all experiments are available at https://github.com/echarvel3/reskmer_data and Zenodo under a CC BY 4.0 license. All empirical datasets analyzed in this study were obtained from public repositories (see Additional file 1: Tables S1 and S2 and Methods: “Biological data pre-processing” section for relevant accession numbers).
