# Supplementary material for: ReSkmer: modeling repeats allows k-mer-based alignment-free methods to calculate population genomic distances
Source: Genome Biol. 2026 May 23;27:233. doi: 10.1186/s13059-026-04108-9 (PMC13386924; doi:10.1186/s13059-026-04108-9)
Supplement: Supplementary file 1 — Additional file 1: Supplementary Tables S1-S3, Supplementary Figures S1-21, and Supplementary Notes A1-A4 containing mathematical derivations and algorithmic details [71]. [file 13059_2026_4108_MOESM1_ESM.pdf]

Supplementary Figures and Tables

Species	Assembly	Genome Size	UR
Selaginella moellendorffii	GCF_000143415.4	208,216,466	0.42
Chondrus crispus	GCF_000350225.1	103,988,016	0.51
Ostrea edulis	GCF_947568905.1*	98,251,904	0.64
Nemopilema nomurai	GCA_003864495.1	210,407,387	0.71
Brachionus plicatilis	GCA_010279815.1	101,125,688	0.85
Hirudo medicinalis	GCA_011800805.1	155,214,494	0.90

Table S1: **Assemblies used in simulations.** Only a single scaffold (NC_079166.1) of the *O. edulis* assembly is represented in our simulations to provide a uniqueness ratio in between other genomes.

Species	NCBI Accession
P. inortata	PRJNA263122
C. fusca	PRJNA263122
G. conirostris	PRJNA263122
O. maximowiczii	PRJNA1224281
O. aries	PRJNA797957
A. cerana	PRJNA1054499
S. sinica	PRJNA936440
D. melanogaster	PRJNA268677; PRJNA30085; SRP006733
A. mellifera	PRJNA363032

Table S2: **NCBI Bioprojects.** Listed are the NCBI accession numbers for all empirical datasets in this study. Note that for some analyses, only a subset of populations were used from each publication.

Method	Skmer Reference	Spectrum Creation
Skmer	3m 15.363s	*NA*
ReSkmer-noref	2m 56.723s	2m 31.169s
ReSkmer-ref	2m 45.697s	16m 35.023s

Table S3: **Running time per sample comparison across methods.** Comparison of running times between Skmer methods. Skmer reference running time reflects how long it takes to process one sample. Spectrum creation reflects how long processing of a reference takes independently of skmer. These running times are based on a single *C. crispus* 2× skim. Organisms with larger genomes or sequences with higher coverage will take longer than what is shown here. While ReSkmer-noref, spectrum estimation has to be run per sample, if an assembly is available, ReSkmer-ref spectrum only needs to be run once on the reference genome, regardless of the number of samples analyzed. Times shown here are based on multithreading with 5 threads and on an *Intel(R) Xeon(R) Silver 4110 CPU @ 2.10GHz.*

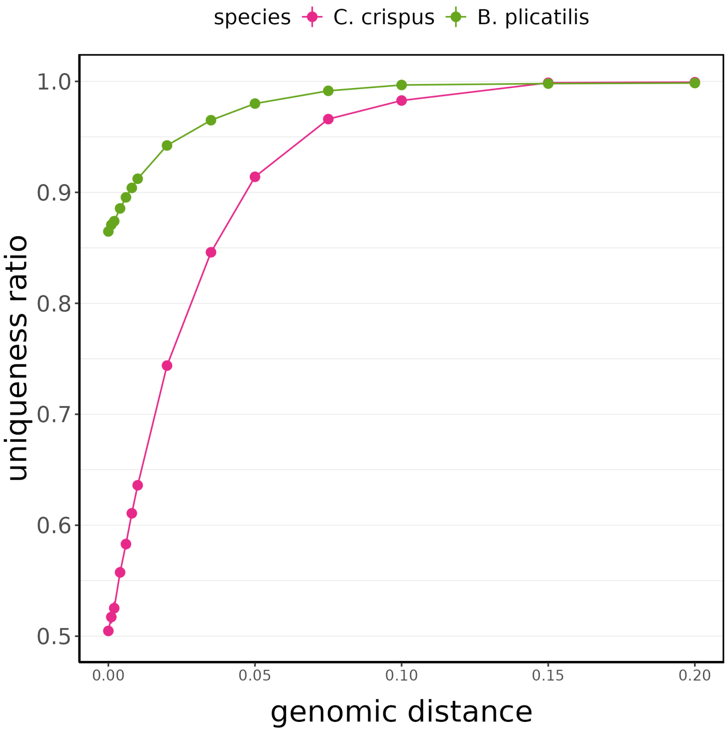

Fig S1: **Parent-child model breaks repeats.** In the parent-child model of evolution, as distance increases (x-axis), the uniqueness ratio increases because SNPs with high probability break repeats and create new ones with negligible probability. Note, however, that at lower distances (e.g., $\leq 1\%$, these changes are not dramatic.

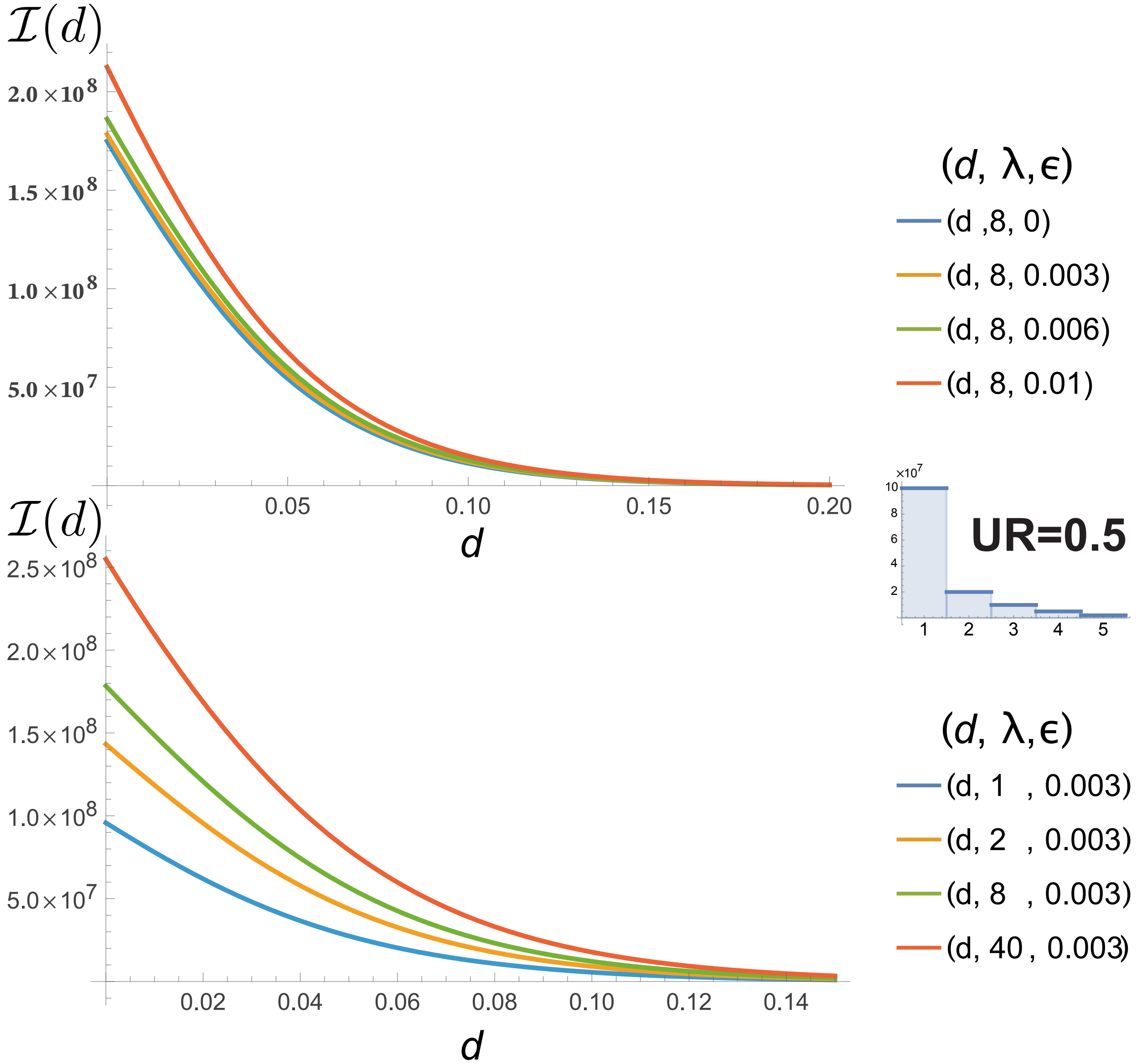

Fig S2: **Monotonicity.** Plots show the distance (x-axis) Eq. (5) outputs given an input intersection $\mathcal{I}$ (y-axis) for a highly repetitive genome (UR= 0.5). Top plot varies the error rate (ϵ) parameter ranging from $0.0 - 0.01$, while the bottom plot varies k-mer coverage (λ) ranging from $1\times - 40\times$. Note that for no intersection are there multiple corresponding distances.

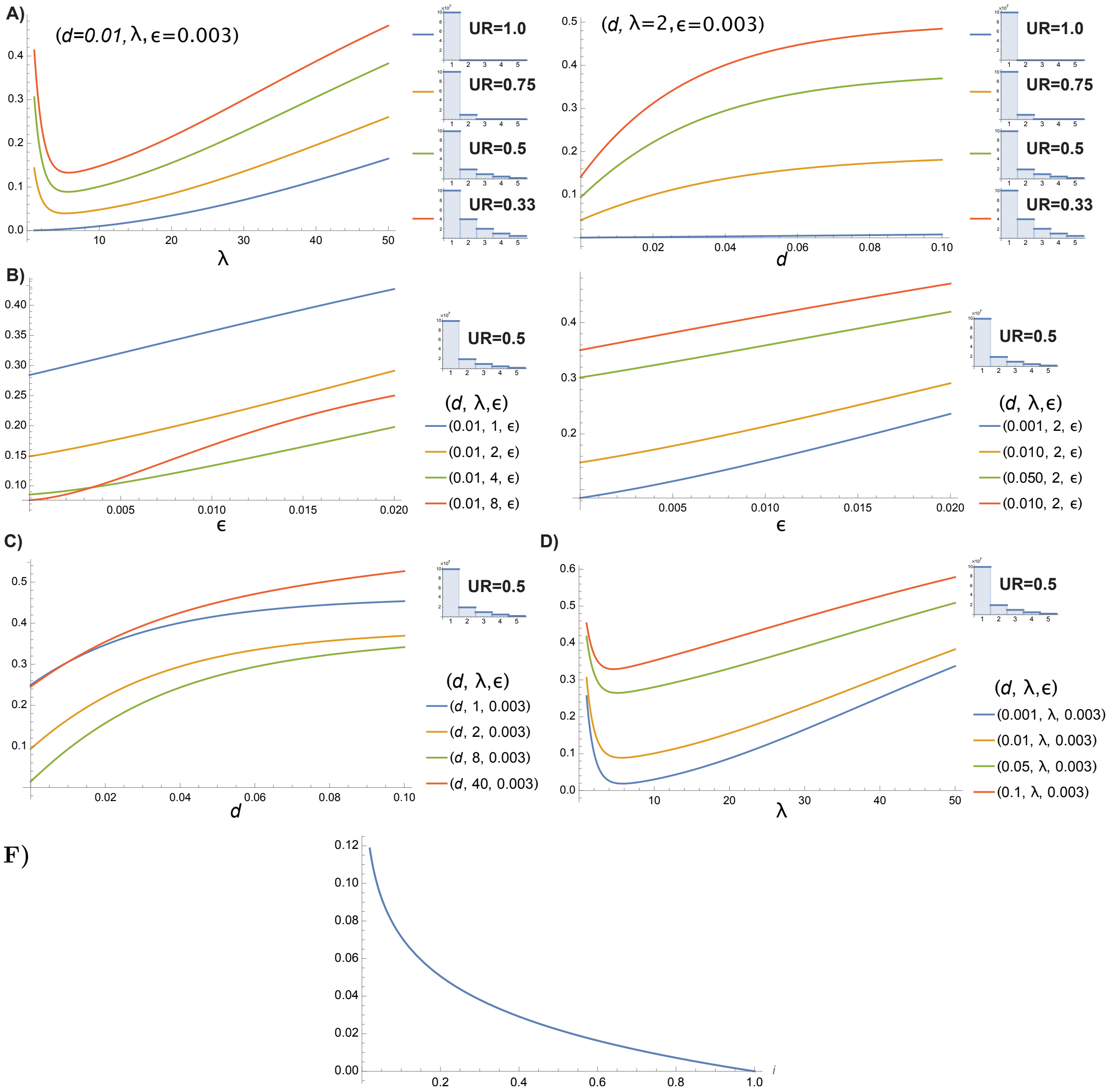

Fig S3: **Analytical comparison of Skmer and ReSkmer.** A-E: The y-axis shows $\frac{|I^{s2}|-|I^{s1}|}{|I^{s2}|}$ where $s1$ uses Skmer Eq. (1) and $s2$ uses ReSkmer Eq. (3). Each panel changes a different parameter (x-axis), fixing other parameters to one of four alternative values (side legends). (A) Changes λ (left) and d (right) for four repeat spectra. UR gives the uniqueness ratio; the first spectrum (blue) is repeat-free. (B-D) Fix UR=0.5 and vary (B) ϵ, (C) d, and (D) λ. F) The intersection (x-axis, shown as the probability of each k-mer being in the intersection) and distance (y-axis) have a non-linear relationship; relatively smaller errors in intersection size can have larger impacts on the estimated distance. **Observations:** Divergence reaches above 50% with highly repetitive genomes and increases with lower UR, higher error (ϵ), and distance (d). The impact of coverage (λ) is more subtle, with low coverage and high coverage being impacted the most. When UR is high, divergence is low with low covrage (e.g., $< 6\times$) but increases with high coverage (e.g., > 10) due to the introduction of erroneous k-mers that appear multiple times. Skmer addressed this issue by removing singletons for coverage above $4\times$. With repetitive genomes, Eq. (1) underestimates the intersection size compared to Eq. (3) even at lower coverage, and the underestimation exacerbates with lower UR, higher distance (d), and higher error rates (ϵ) but have a non-monotonic relationship with coverage λ (B-D). Underestimation is a major problem for low coverage (e.g., $< 3\times$), quickly drops for medium coverage ($3-10\times$), and rises again for higher coverage values (e.g., $> 10\times$).

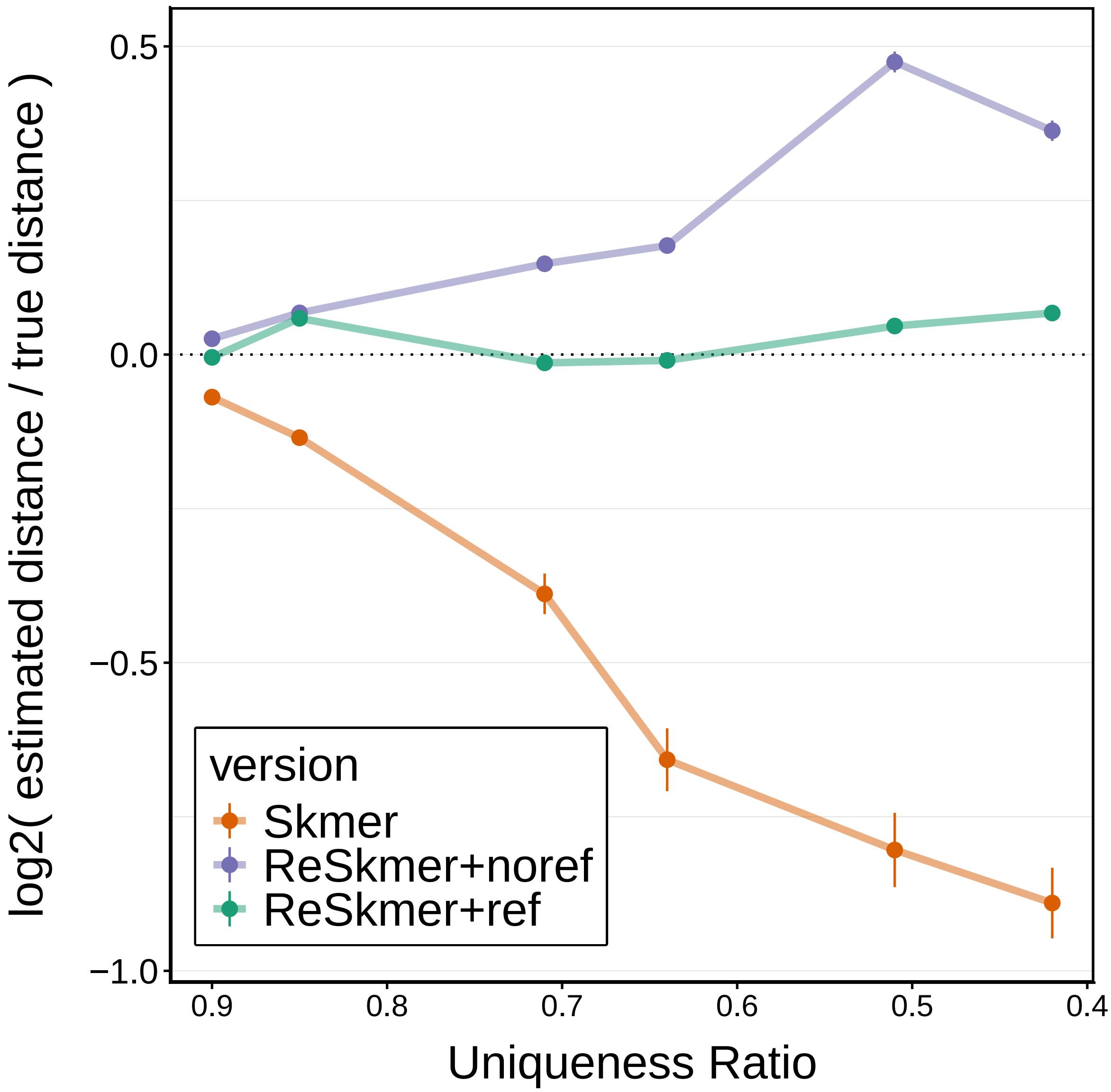

Fig S4: **Skmer log-fold error increases with repetitiveness.** Alignment-free distance estimation accuracy (y-axis) in simulated data is plotted against decreasing uniqueness ratio (increasing repetitiveness). Each data point represents the average log-fold error across all distances, coverages, and replicates for a genome at that UR.

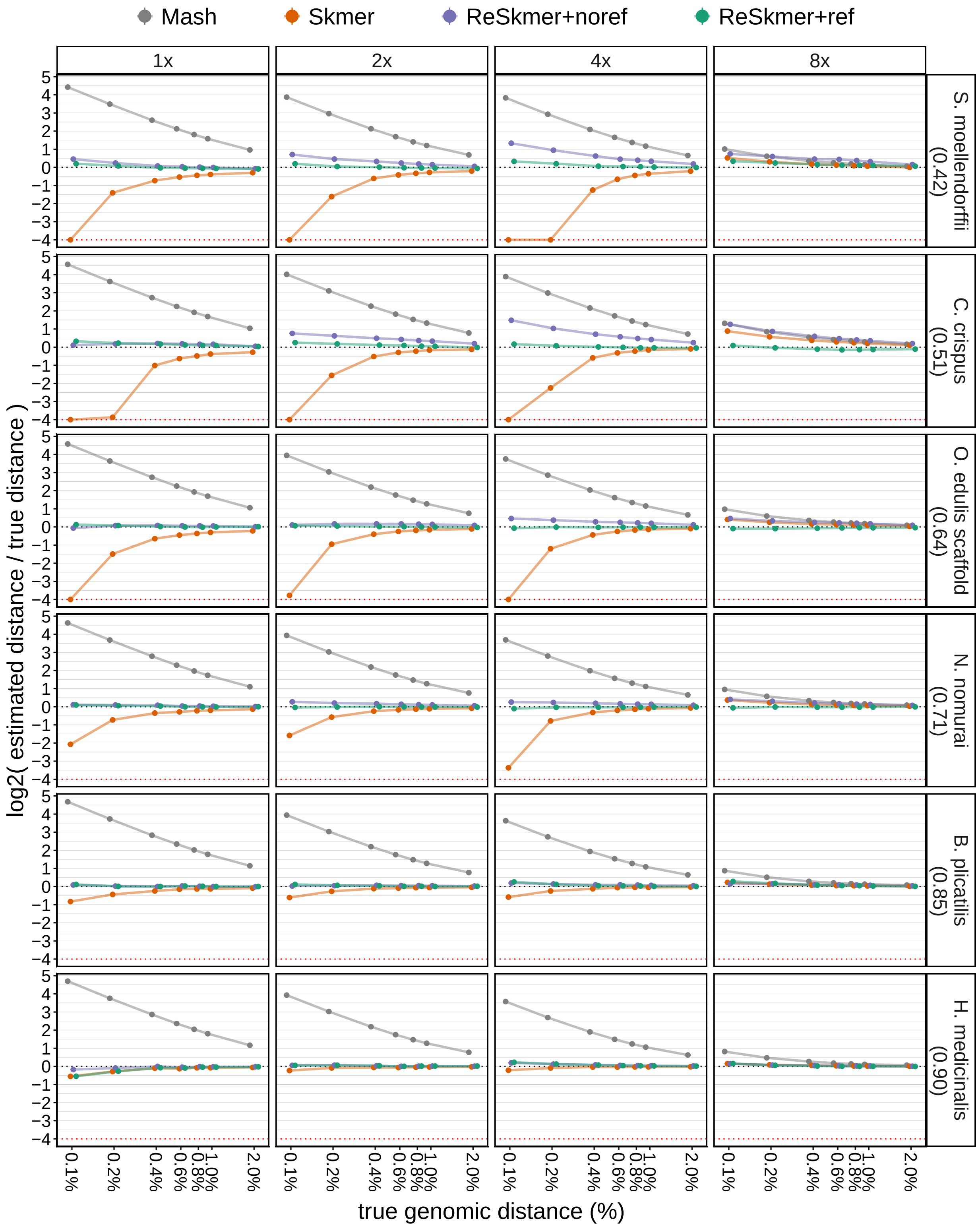

Fig S5: **Error in population-level distance estimation across all genomes.** Comparison the accuracy of Skmer and ReSkmer in simulated sequencing runs across a range of coverages (top strip) and the full range of species (right strip, showing uniqueness ratio parenthetically). The mean log-fold error across replicates, $\log_2(d_{\text{estimated}}/d_{\text{true}})$, ($y$-axis) is plotted against genomic distance (x-axis). When $d_{\text{estimated}} = 0$, instead of $-\infty$, we show $y = -4$ (the red dotted line).

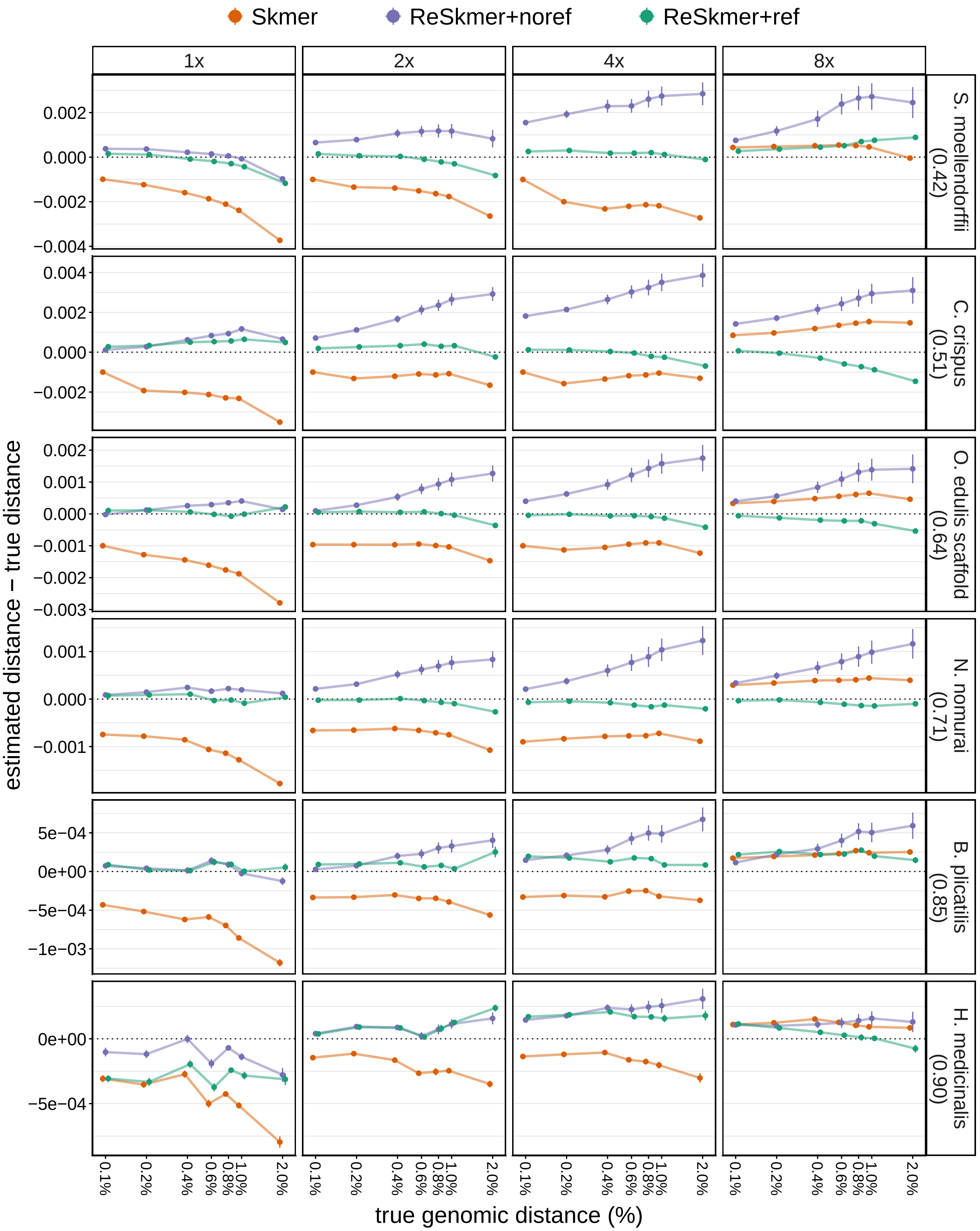

Fig S6: **Absolute difference between estimated and true distances.** Comparing the accuracy of Skmer and ReSkmer in simulated sequencing runs across a range of coverages (top strip) and species (right strip, showing uniqueness ratio parenthetically). Similar to Figure S5 the x-axis represent simulated distances, but here we show the absolute error $d_{estimated} - d_{true}$ on the y-axis (without log). Mash is not included here due to its large overestimation in 1–4× coverage.

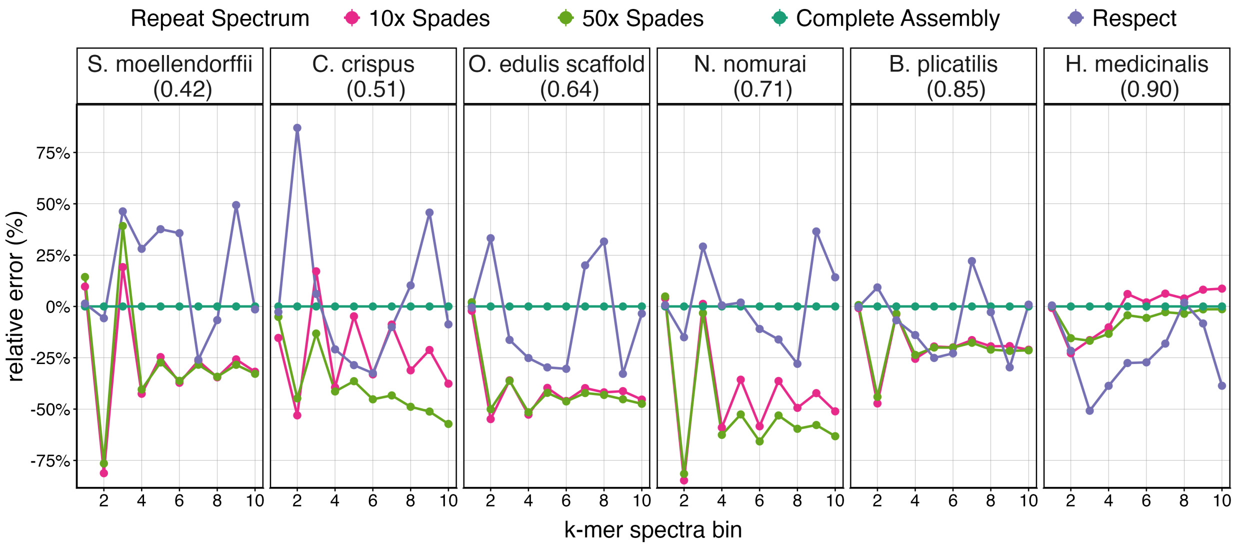

Fig S7: **Percent error in r_i estimation using Spades assemblies.** Accuracy of estimated repeat spectra ($\hat{r}_i$) computed using Jellyfish from Spades assemblies (Illumina reads, 10× and 50× coverages) or using RESPECT from a 4× genome skim. We show $\hat{r}_i/r_i - 1$ for $i \leq 10$ (x axis). "Complete Assembly" refers to the finished genome which provides the true spectrum (r_i).

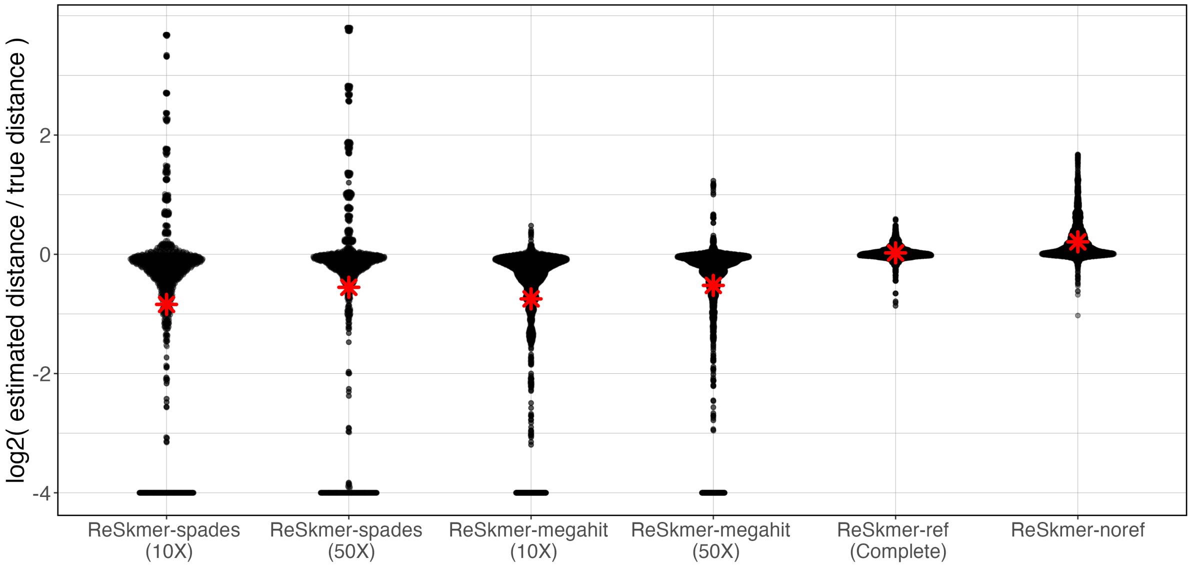

Fig S8: **Log-fold error in distance estimation using different repeat spectra.** The effect of repeat spectra estimated using different assemblers on the log-fold error in ReSkmer distance estimation across all genomes, coverages, and distances. On the y-axis, log-fold error of each measure of d is plotted. The asterisk represents the mean for each method.

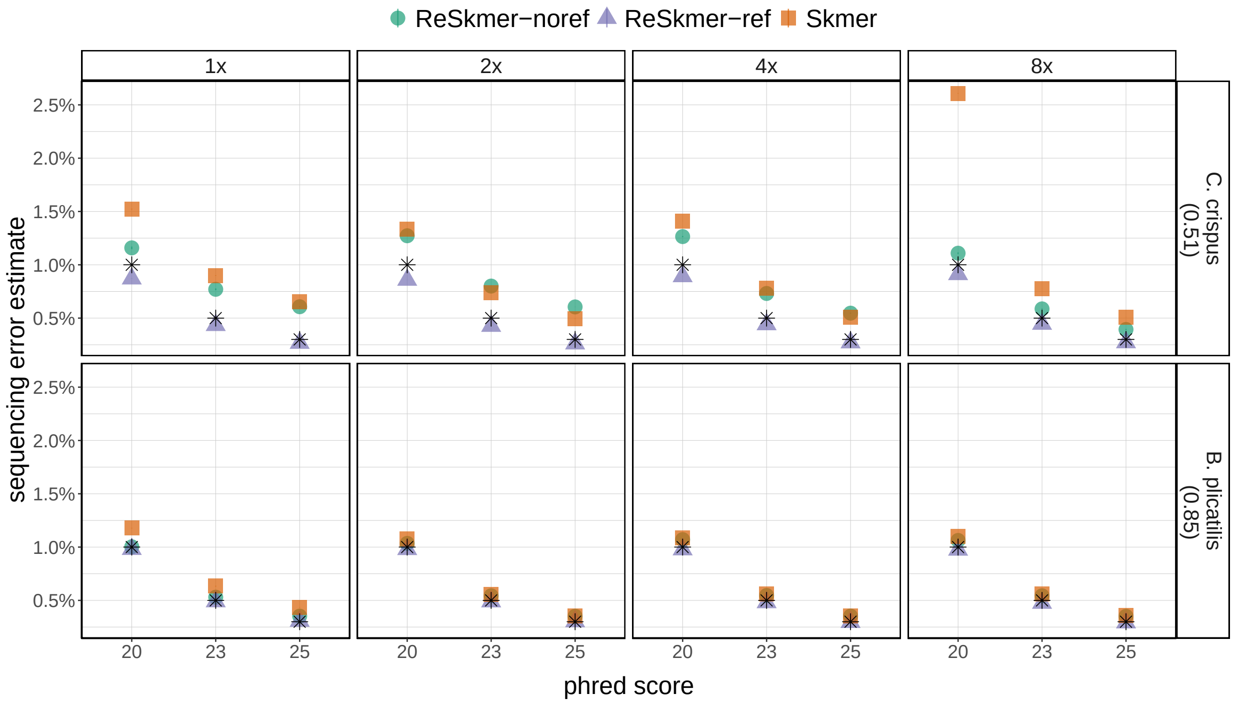

Fig S9: **Sequencing Error Rate (ϵ) Estimation.** Comparison of the accuracy of different methods when estimating sequencing error rate. Each color represents a mean estimate of sequencing error in % (*y-axis*) skims where d=0%. Different error rates are found in the axis as phred quality scores. Asterisks represent the true value of the error rate in %. Here we show results for two different genomes, a high repeat genome (*C. crispus*) and a low repeat genome (*B. plicatilis*).

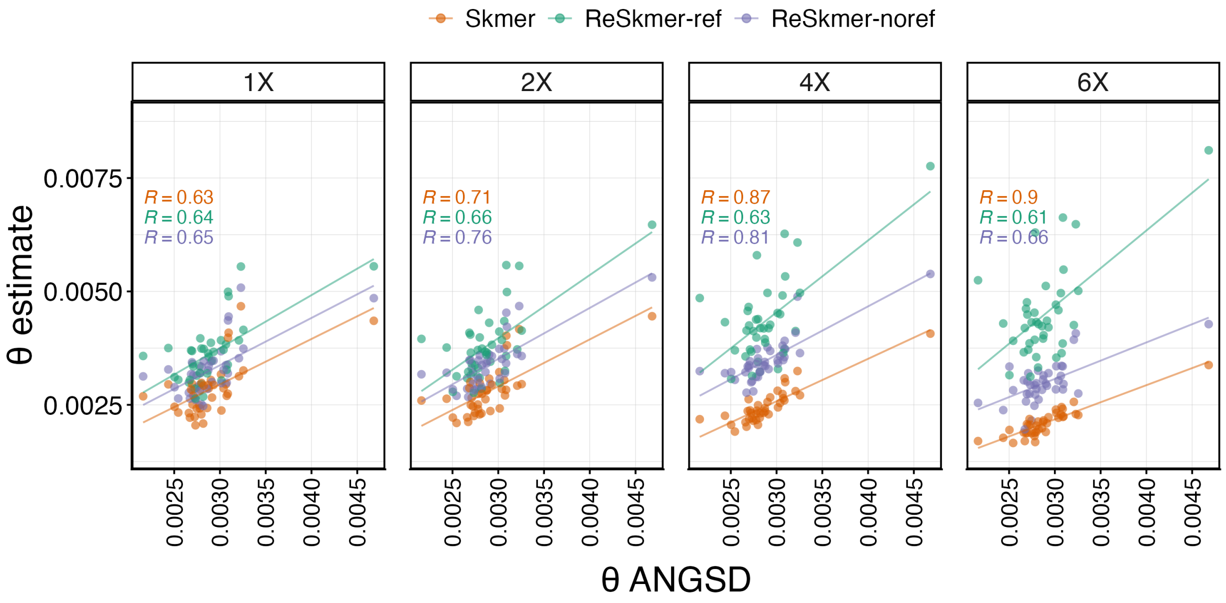

Fig S10: **ReSkmer correlates with ANGSD in *Apis mellifera*.** Pearson correlation results for Skmer estimates of θ (y-axis) with those produced by ANGSD (x-axis) at different coverages (top strip). Pearson correlation coefficients are found in the top left corner of each panel.

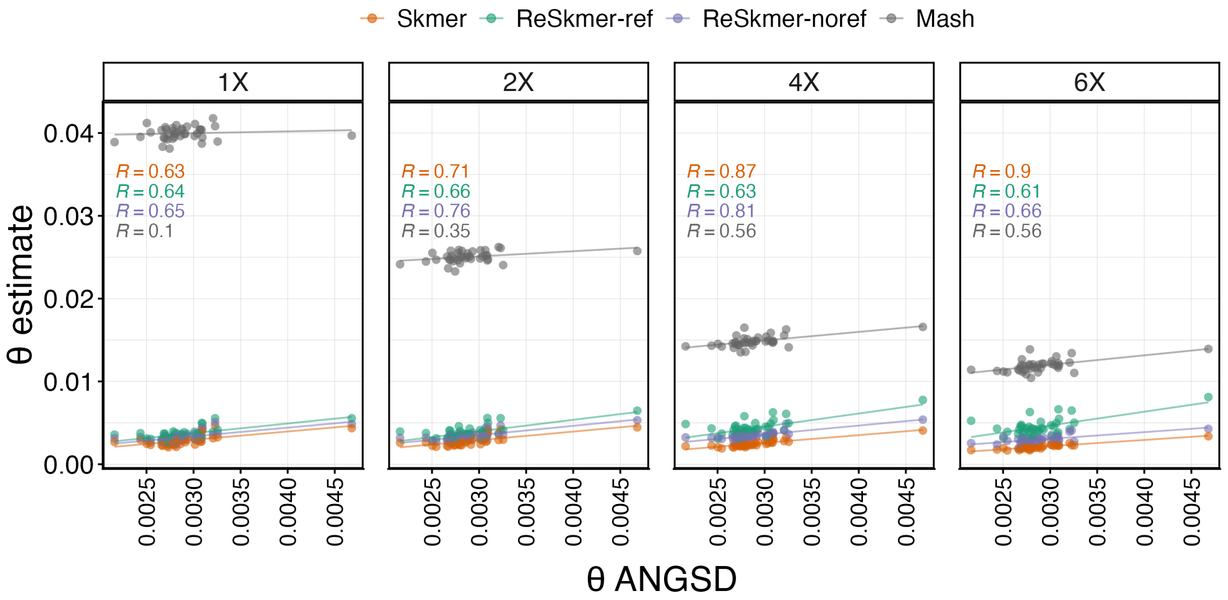

Fig S11: **Comparison of ReSkmer and Mash correlation with ANGSD in Honey Bees.** Correlation plots of different Skmer estimates of θ (y-axis) compared to those produced by ANGSD (x-axis) at different coverages (top strip). Mash (in grey) produces very large estimates of θ and is outperformed by ReSkmer at low coverages. Pearson correlation coefficients are found in the top bottom corner of each panel.

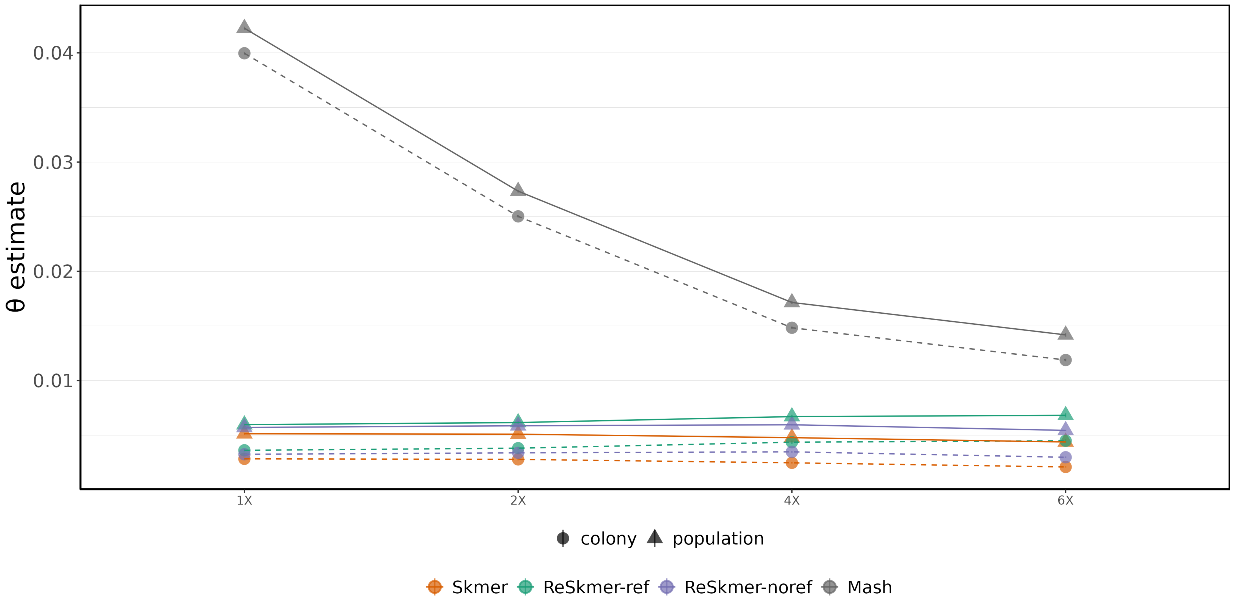

Fig S12: **Comparison of ReSkmer and Mash stability across coverage in *Apis mellifera*.** Values of θ (y-axis) for both populations (solid lines) and colonies (dashed lines) estimated by Mash have a much greater range across coverage (x-axis) compared to those produced by ReSkmer. Traditional estimates of *Apis mellifera* θ are in the range of $0.001 - 0.003$[1].

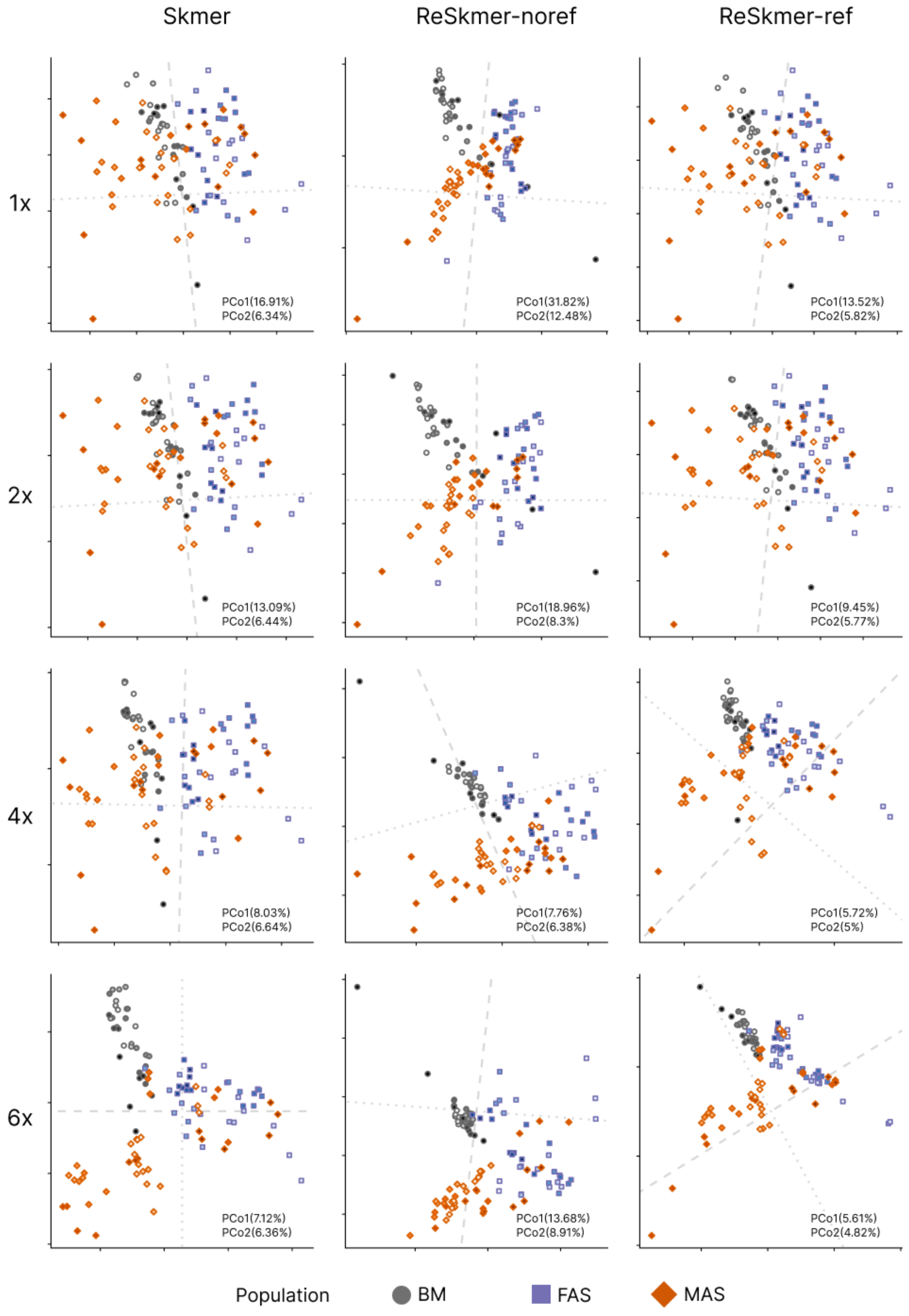

Fig S13: **MDS Clustering of *Apis mellifera* Populations.** PCo1 and PCo2 of PCoA analysis of distance matrices produced by Skmer. Each of the three honey bee populations have been differentiated by color, and members of the same hive have the same shade of their population color. Plots have been rotated with the procrustes method to align populations. The direction of the original axes are denoted by a dashed line (PCo1) and dotted line (PCo2).

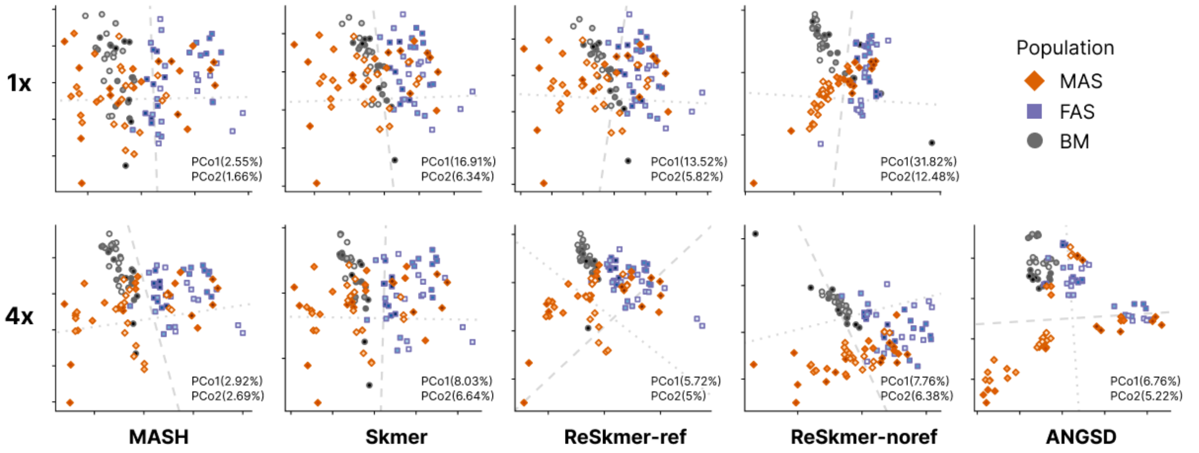

Fig S14: **Comparison of *Apis mellifera* population structure of all methods.** PCo1 and PCo2 of PCoA analysis of distance matrices produced by k-mer-based methods are compared for 33× clustering with ANGSD. Plots have been rotated with procrustes method (dashed line PCo1 axis, dotted line PCo2 axis). All methods, including ANGSD, show some degree of overlap between populations.

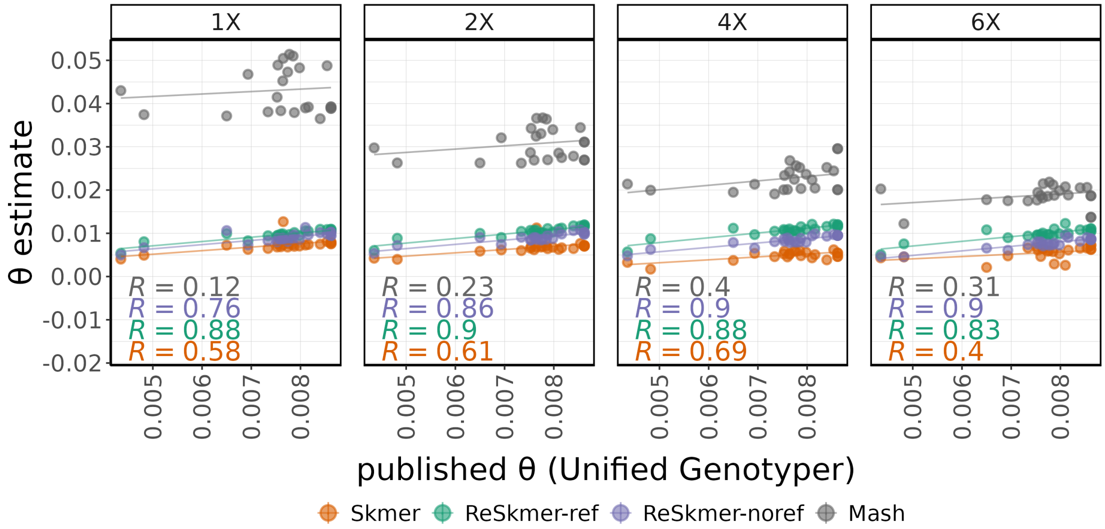

Fig S15: **Comparing Mash and ReSkmer vs. Unified Genotyper θ estimates in *Drosophila*.** Correlation plots of different Skmer estimates of θ (y-axis) compared to those published in the original *Drosophila* study (x-axis). Mash (in grey) again produces very large estimates of θ and is outperformed by ReSkmer at all coverages and does does not perform better at higher coverages. Pearson correlation values for each method are found in the lower left corner for each coverage panel.

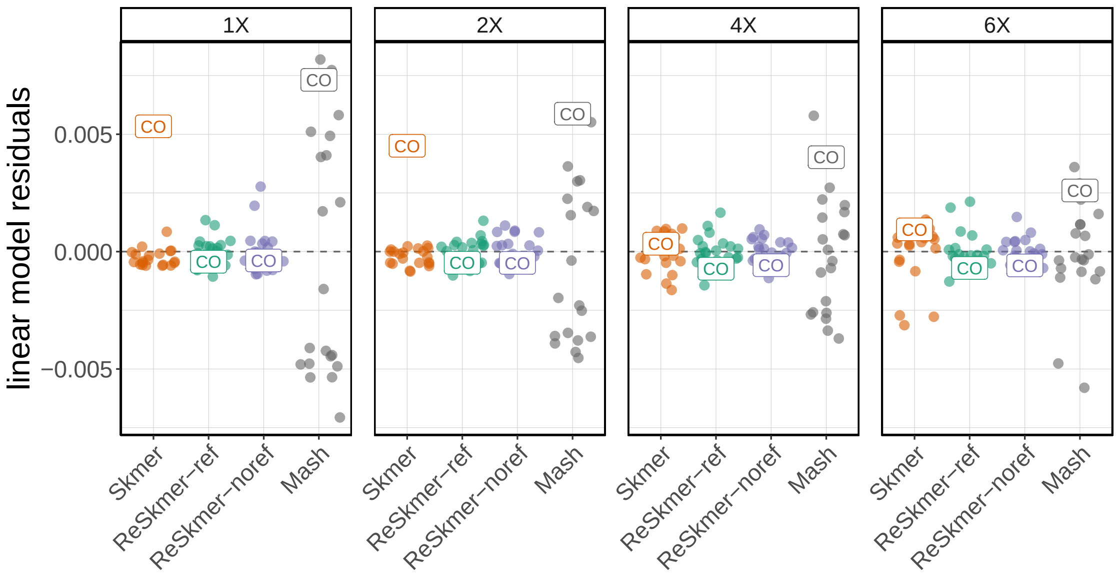

Fig S16: **Distribution of ReSkmer vs. Unified Genotyper Linear Model Residuals in *Drosophila melanogaster*.** Linear models of the correlation between k-mer based methods (x-axis) estimates of θ and those produced by Unified Genotyper are created and their residuals plotted (y-axis) The "CO" population is emphasized as an outlier in low-coverage Skmer analyses. When removed, correlation for all methods becomes more or less equal across methods at low coverages (1-2×). Outlier-free correlations ordered by ReSkmer-noref, ReSkmer-ref, and Skmer: 1×: (0.76, 0.88, 0.93); 2×: (0.87, 0.90, 0.92); 4×: (0.90,0.88, 0.69); 6×: (0.91, 0.83, 0.41).

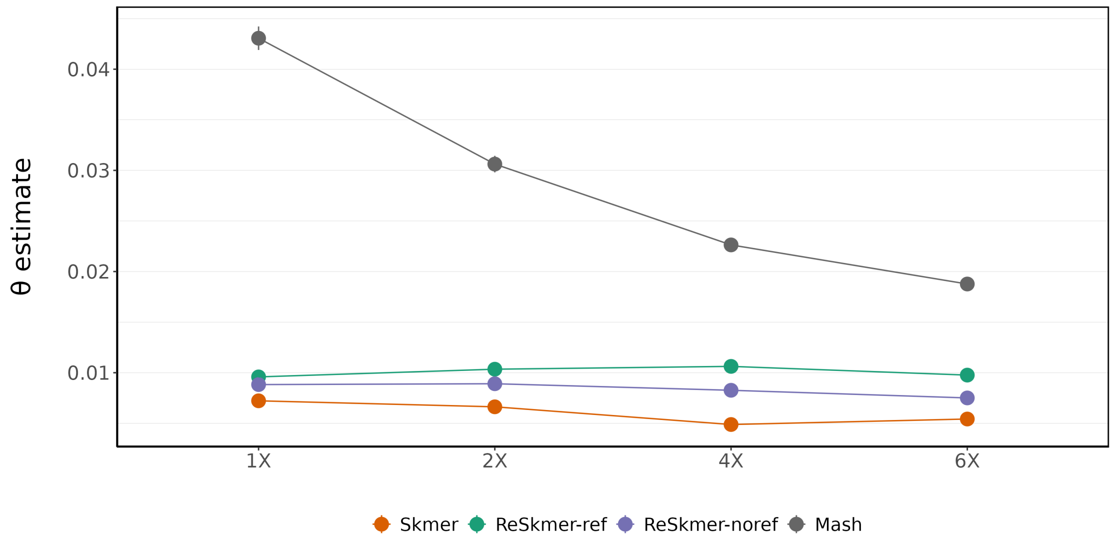

Fig S17: **Comparison of ReSkmer and Mash stability across coverage in *Drosophila melanogaster*.** Estimates of population θ in fruit flies (y-axis) are more unstable across coverage (x-axis) in Mash compared to other methods.

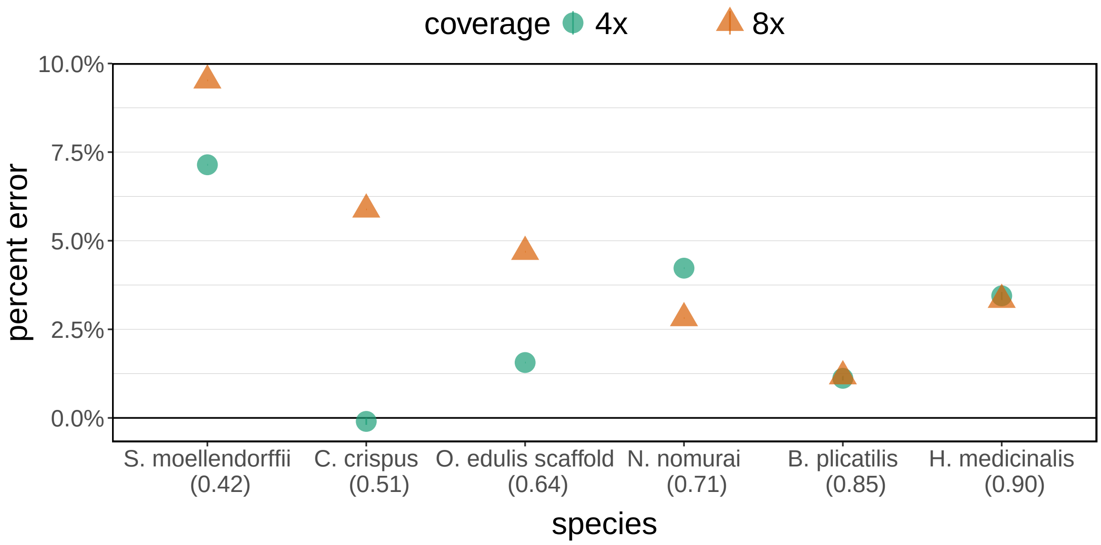

Fig S18: **RESPECT accuracy at different coverages.** Percent error (y-axis) of RESPECT estimates of uniqueness ratio for different genomes (x-axis) ordered from most repetitive to least repetitive. Using $4\times$ coverage is more or just as accurate than $8\times$ in most cases (*N. nomurai* being the exception).

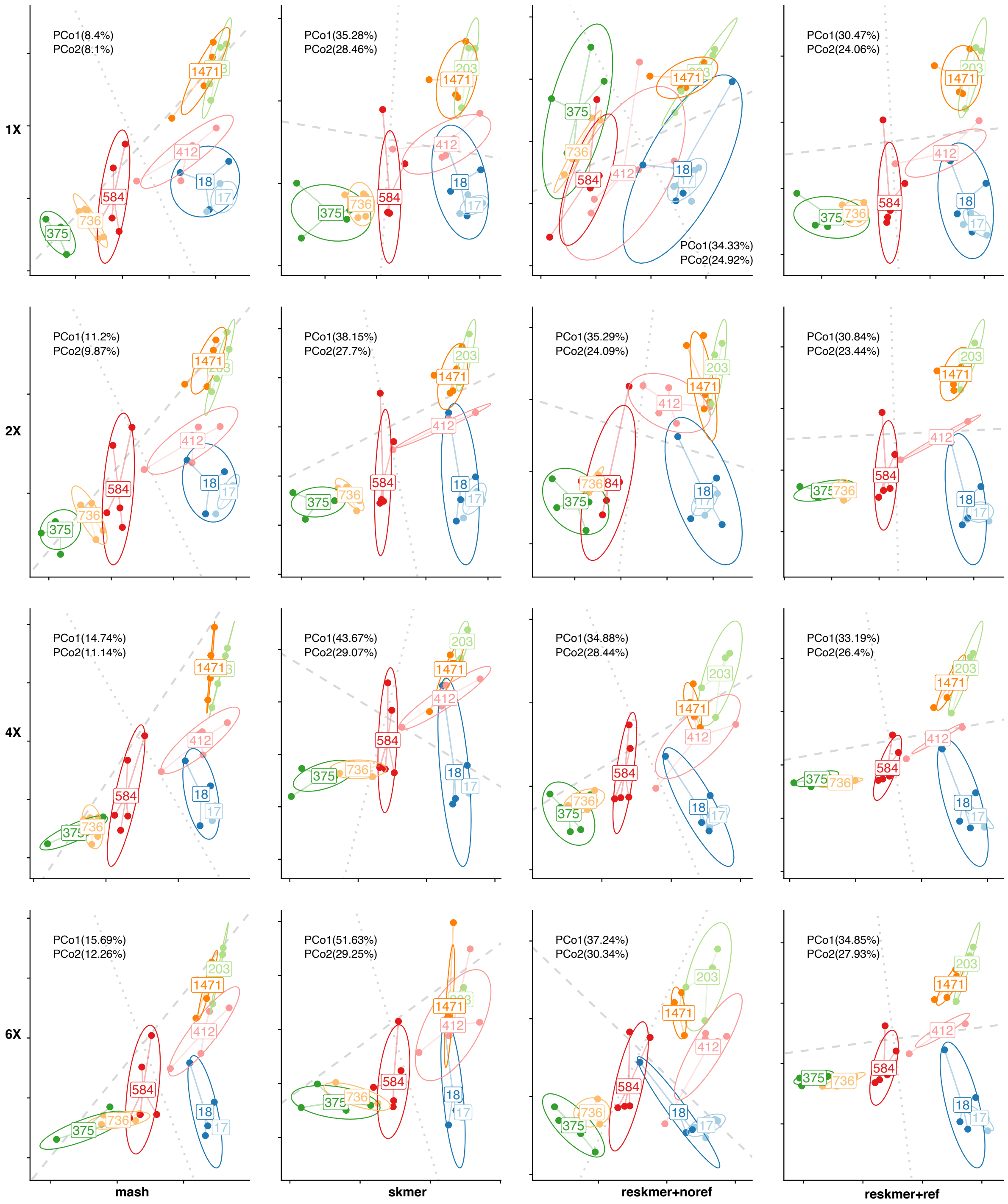

Fig S19: **Clustering of *Enterococcus faecium*.** We reanalyze a dataset with sequences originating from multiple *Enerococcus faecium* sequence types (STs)[2]. For this dataset, we run human read removal with Kraken2. We perform PCoA on resulting distance matrices and plot the first two principal coordinates, PCo1 (dashed line) and PCo2 (dotted). We then observe which methods better separate different STs (Sequence Types). Rows correspond to coverage and columns to different methods. Points represents organisms colored by ST. Plots have been rotated with procrustes. GCF_009734005.1 is used as reference. While Mash is able to cluster ST 375 and 736, it struggles to cluster 735 and 584. Inversely, both Skmer and ReSkmer+ref fail to distinguish 736 from 375, but clearly separate 584 and 736. At 2× and beyond, ReSkmer+ref is the only method able to separate ST 412 from 18. At 6×, ReSkmer+ref becomes the only method that separates all STs, except for 18 and 17, which no method separates.

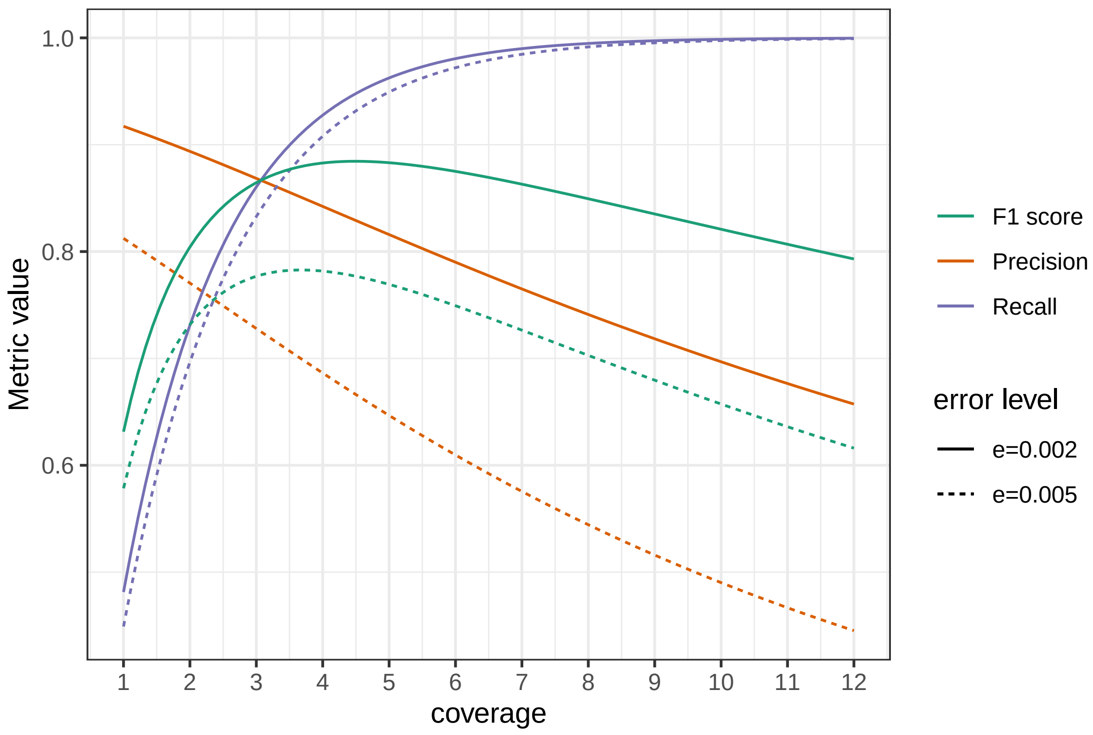

Fig S20: **Theoretical impact of coverage on the proportion of k-mers that are erroneous.** We define the following metrics in a natural way. TP: any k-mer in a genome skim that is in the genome. FN: any k-mer of a genome that is not in a genome skim. FP: any k-mer in a genome skim that is not in the genome (due to errors). These readily define precision, recall, and F1 score in the standard way. We show these three metrics for a range of coverage values (λ) and two error levels (ϵ). Note how the F1 measure, which combines precision and recall, attains its optimum value somewhere between 2 and 5X coverage. We calculate all three measures in expectation, using these equations. FP: $L\lambda\left(1-(1-\epsilon)^k\right)$. FN: $Le^{-\lambda(1-\epsilon)^k}$. TP: $L(1-e^{-\lambda(1-\epsilon)^k})$. The genome length L cancels out and is not consequential.

Supplementary Text

A Method Details

A.1 Parameter Dependency

The pairwise dependencies between these parameters are shown in Fig. S21. Over-, or underestimation of the parameters could result in over- or underestimation of the size of the intersection. As a challenging example, consider a case where we overestimated error $\epsilon^{(i)}$. The overestimation of ϵ is correlated with an underestimation of $\xi^{(i)}, \eta^{(i)}$, which in turn reduces $|I_0|$. However, overestimating $\epsilon^{(i)}$ increases $\mathbb{E}[n_1^{(2)}]$, and $\mathbb{E}[n_1^{(2)}]$. This in turn increases $\mathbb{E}[|I_1|]$. Thus, the overestimation of error has a complicated outcome.

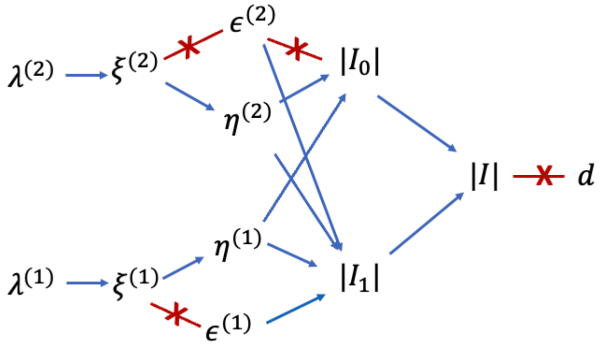

Fig S21: Dependencies between pairs of parameters.

A.2 Adjusting RESPECT results

When spectrum is obtained from RESPECT, we apply to adjustments to its output.

Genome length adjustment. In the RESPECT output, $\sum_i ir_i$ does not necessarily match its estimate of the genome length L, as one would expect. In the original publication[3], estimates of genome lengths and r_1 were more accurate than its estimates of repeat spectrum ($r_i, i \geq 2$ values). In ReSkmer, we need r_i values, and we need $\sum_i ir_i$ to sum up the genome length. To deal with this discrepancy, instead of using r_i values, we instead use the genome length estimate L and r_1 from RESPECT, and adjust other values as:

$$r'_i = \begin{cases} r_1 & i = 1 \\ r_i \frac{L - r_1}{(\sum_i ir_i) - r_1} & i > 1 \end{cases}$$

We can confirm that

$$\sum_i ir'_i = r_1 + \sum_{i>1} ir_i \frac{L - r_1}{(\sum_j jr_j) - r_1} = r_1 + \frac{L - r_1}{(\sum_j jr_j) - r_1} \sum_{i>1} ir_i = r_1 + L - r_1 = L$$

Smoothing of r_i values. We noted that unlike spectra obtained from genomes, RESPECT output can often lack smoothness in log space. See Figure below.

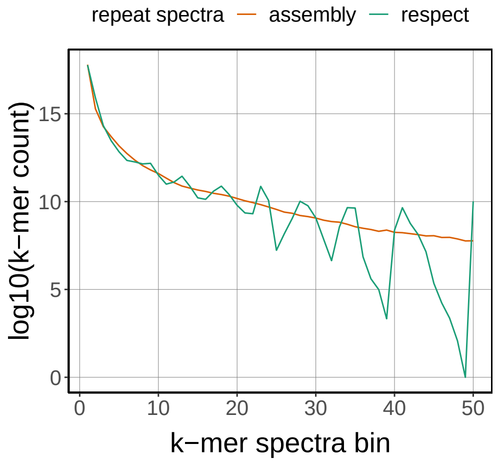

Fig S22: **RESPECT Lacks Smoothness in Log Space (*C. crispus*).**

To smooth a respect input, we first obtain an approximation of the slope of the repeat spectrum with the following equation:

$$\text{slope}_{sample} = \left(\log_{10}(r_m + p) - \frac{\log_{10}(r_{M-3} + p) + \log_{10}(r_{M-2} + p)}{2} \right) \times \frac{1}{M - m}$$

where p is a pseudo-count to avoid 0, $M = 50$ is the maximum bin, and $m = 10$ is the start point for soothing. We begin to calculate our slope at bin 10 because bins before that point are not linear in log space and are smooth. Then we smooth k-mers as follows:

Algorithm 1 RESPECT Smoothing

---

for each sample s in RESPECT Spectrum Input **do**
  for 5000 randomly generated integers, i, within range between (m, M) **do**
    if difference between $|\log_{10}(r_i) - \log_{10}(r_{i+1})|$ is larger than slope$_{sample}$ **then**
      if $r_i > r_{i+1}$ **then**
        $y = \frac{ir_{(i)} + (3i+1)r_{(i+1)}}{2(2i+1)}$
      else
        $y = \frac{(i+2)r_{(i+1)} + 3ir_{(i)}}{2(2i+1)}$
      $r_i = \frac{ir_{(i)} + r_{(i+1)} - y(i+1)}{i}$
      $r_{i+1} = y$
      stop counter = 0
    else
      stop counter += 1
    if stop counter reaches 50 **then**
      break (smoothing for sample s finished)

A.3 Derivation of $\mathbb{E}[e^{-\frac{M^{(2)}}{3k}}]$

We investigate one of the i copies of a k-mer, denoted by u. Let Y indicate the number of mutations of u in the second genome. Then,

$$\begin{aligned}\mathbb{E}[e^{-\frac{M^{(2)}}{3k}}] &= \mathbb{E}[\mathbb{E}[e^{-\frac{M^{(2)}}{3k}}|Y]] \\ &= \underbrace{P(Y=0)\mathbb{E}[e^{-\frac{M^{(2)}}{3k}}|Y=0]}_{n^*_{21}} + \underbrace{P(Y=1)\mathbb{E}[e^{-\frac{M^{(2)}}{3k}}|Y=1]}_{n^*_{22}} + \underbrace{P(Y>1)\mathbb{E}[e^{-\frac{M^{(2)}}{3k}}|Y>1]}_{n^*_{23}}\end{aligned} \tag{1}$$

When $Y_j = 0$, clearly $M^{(2)}$ behaves similarly to $M^{(1)}$. Thus, recalling Eq. (8),

$$n^*_{21} = (1-d)^k e^{\lambda^{(2)}\epsilon(1-\epsilon)^{k-1}k\left(e^{-1/3k}-1\right)} = (1-d)^k e^{-b\lambda^{(2)}\epsilon(1-\epsilon)^{k-1}} .$$

Now, let's turn to n^*_{22}. Let $C \sim \text{Poisson}(\lambda^{(2)})$ be coverage of u. Let $\beta = 1-(1-\epsilon^{(2)})^k$. Then,

$$P(M^{(2)}|Y=1,C) = \begin{cases} 1-(\beta)^C & M^{(2)}=1 \\ (\beta)^C & M^{(2)}=0 \\ 0 & \text{else} \end{cases} .$$

Therefore, the probability-generating function of $M^{(2)}|(Y=1,C)$ is $z\left(1-\beta^C\right)+\beta^C$. Thus,

$$\mathbb{E}[e^{-\frac{M^{(2)}}{3k}}|Y=1,C] = e^{-1/3k}\left(1-\beta^C\right)+\beta^C = \beta^C(1-e^{-1/3k})+e^{-1/3k}$$

and

$$\begin{aligned}\mathbb{E}[e^{-\frac{M^{(2)}}{3k}}|Y=1] &= \mathbb{E}[\mathbb{E}[e^{-\frac{M^{(2)}}{3k}}|Y=1,C]] = \mathbb{E}[\beta^C](1-e^{-1/3k})+e^{-1/3k} \\ &= e^{\lambda^{(2)}(\beta-1)}(1-e^{-1/3k})+e^{-1/3k} = e^{-\lambda^{(2)}(1-\epsilon^{(2)})^k}(1-e^{-1/3k})+e^{-1/3k}\end{aligned}$$

where we use the probability-generating function of the Poisson distribution ($\mathbb{E}[z^x] = e^{\lambda(z-1)}$).

Finally, for n^*_{23}, if two or more mutations fall on u, we get a 1-neighbor only if an error cancels one of the mutations. Ignoring this probability,

$$n_{23}^* = \Big(1-(1-d)^k - kd(1-d)^{k-1}\Big)\, e^0 \; .$$

Plugging everything back into Eq. (S1) and recalling $b = k(1-e^{-1/3k}) \approx 1/3$, we get:

$$\begin{aligned}
&\mathbb{E}[e^{-\frac{n_2^*}{3k}}] = \mathbb{E}[\mathbb{E}[e^{-\frac{n_2^*}{3k}}|Y]] = \\
&\quad (1-d)^k e^{-b\lambda^{(2)}\epsilon(1-\epsilon)^{k-1}} + d(1-d)^{k-1}\Big(be^{-\lambda^{(2)}(1-\epsilon^{(2)})^k} - b + k\Big) + \Big(1-(1-d)^k - kd(1-d)^{k-1}\Big) = \\
&\quad (1-d)^k e^{-b\lambda^{(2)}\epsilon(1-\epsilon)^{k-1}} + d(1-d)^{k-1}b\Big(e^{-\lambda^{(2)}(1-\epsilon^{(2)})^k} - 1\Big) + \Big(1-(1-d)^k\Big) = \\
&\quad (1-d)^k e^{-b\lambda^{(2)}\epsilon(1-\epsilon)^{k-1}} - bd(1-d)^{k-1}\eta^{(2)} + \Big(1-(1-d)^k\Big)
\end{aligned}$$

A.4 Details of ξ optimization

Choosing H_2. For $j \in [2, 10 \times \lceil\lambda\rceil)$, we set $H_2 = \lfloor\lambda\rfloor + j$, bounding it from below by 5 and from above at the maximum value for which we have the spectrum calculated (50 by default). After solving the optimization problem using each H_2, we calculate the mean weighted least square error for each H_2 using

$$\frac{1}{H_2 - H_1 + 1} \sum_{h=H_1}^{H_2} \frac{1}{o_h} \left(o_h - \sum_{i=1}^{m} r_j \left(\frac{e^{-i\xi}(i\xi)^h}{h!} \right) \right)^2$$

and choose the H_2 that minimizes this error.

Optimization bounds: The function

$$\sum_{i=1}^{m} r_j \left(\frac{e^{-i\xi}(i\xi)^h}{h!} \right)$$

used in our optimization is non-monotonic with respect to ξ, as shown using the following Mathematica expression:

```
In[ ]:= r = {0.9, 0.01, 0.02, 0.005, 0};
        ExpOh[ξ_, h_] := r.Table[(Exp[-j ξ] ((j ξ)^h) / (h !)), {j, 5}]
        Plot[{ExpOh[ξ, 3], ExpOh[ξ, 6], ExpOh[ξ, 10], ExpOh[ξ, 14]}, {ξ, 0.1, 24}
          , PlotLegends → "Expressions", AxesLabel → Automatic]
```

Out[ ]=

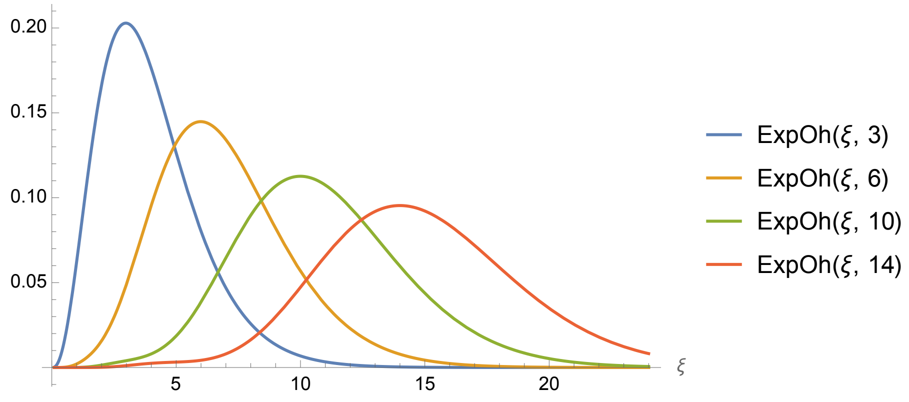

For any observed o_h value on the y-axis, there are two values of ξ that match it. Recall $\xi = \lambda(1-\epsilon)^k$. We can easily see that only $\xi < \lambda$ values will lead to $\epsilon > 0$, and thus, the solution $\xi > \lambda$ is irrelevant to our application. Thus, we need bounds to guide the optimization problem to find sensible ξ values. In practice, assuming that $\epsilon \in [0.0001, 0.03]$, we set the bounds to $[\lambda(1-0.03)^k, \lambda(1-0.0001)^k]$.

References

[1] Gonçalo Espregueira Themudo, Alba Rey-Iglesia, Lucía Robles Tascón, Annette Bruun Jensen, Rute R da Fonseca, and Paula F Campos. Declining genetic diversity of european honeybees along the twentieth century. *Scientific reports*, 10(1):10520, 2020.

[2] Mustapha M Mustapha, Vatsala R Srinivasa, Marissa P Griffith, Shu-Ting Cho, Daniel R Evans, Kady Waggle, Chinelo Ezeonwuka, Daniel J Snyder, Jane W Marsh, Lee H Harrison, et al. Genomic diversity of hospital-acquired infections revealed through prospective whole-genome sequencing-based surveillance. *Msystems*, 7(3):e01384–21, 2022.

[3] Shahab Sarmashghi, Metin Balaban, Eleonora Rachtman, Behrouz Touri, Siavash Mirarab, and Vineet Bafna. Estimating repeat spectra and genome length from low-coverage genome skims with RESPECT. *PLOS Computational Biology*, 17(11):e1009449, November 2021.
